# Direct evidence and quantification of homologous recognition between DNA duplexes

**DOI:** 10.1073/pnas.2530949123

**Published:** 2026-06-04

**Authors:** Andrew Stannard, Ehud Haimov, Jonathan G. Hedley, Yaxuan Xiao, Marco Di Antonio, Gleb Oshanin, Claudia Danilowicz, Mara Prentiss, Lorenzo Di Michele, Alexei A. Kornyshev

**Affiliations:** ^a^ Department of Chemistry, Imperial College London, London W12 0BZ, United Kingdom; ^b^ Department of Chemical Engineering and Biotechnology, University of Cambridge, Cambridge CB3 0HE, United Kingdom; ^c^ School of Mechanical Engineering, Tel Aviv University, Tel Aviv 69978, Israel; ^d^ Department of Engineering Science, University of Oxford, Oxford OX1 3QD, United Kingdom; ^e^ Laboratoire de Physique Théorique de la Matière Condensée (UMR CNRS7600), Sorbonne Université/CNRS, Paris 75252 Paris Cedex 05, France; ^f^ Department of Physics, Harvard University, Cambridge, MA 02138

**Keywords:** DNA, homology recognition, nano-sensor, electrostatic interactions, ion adsorption

## Abstract

Stretches of double-stranded DNA sharing the same sequence can recognize each other in cells. This phenomenon, known as homologous recognition, is essential for DNA recombination and repair. Yet, its mechanism remains debated, with purely physical interactions proposed as a contributing factor. Here, we use a minimal DNA nanosensor to quantify homologous pairwise interactions with exquisite precision. We find that homology enhances the duplex–duplex affinity induced by physiological divalent cations and measure the homology-driven recognition free energy as ∼−0.01kcal/mol per base pair. This affinity substantially enhances coalignment of homologous DNA in the confined geometry of the nanosensor, which mimics physical effects of concentrated biological environments. We introduce a quantitative electrostatic framework that attributes this emergent behavior to coherent charge distributions unique to homologous DNA. Our findings provide compelling evidence in support of purely physical sequence-specific interactions between intact double-stranded DNA, which may bear biological relevance for homologous recombination.

## Significance

Well-understood, sequence-specific interactions between single-stranded (ss) DNA underpin myriad canonical, i.e., base-paired, and noncanonical, e.g., G-quadruplex, structures. Sequence-specific interactions between double-stranded (ds) DNA duplexes, however, are less intuitive because nucleobase identities are hidden within the helical structure, yet these interactions are central to several biological processes. These include homologous recombination, which is responsible for DNA repair and horizontal gene transfer, the initial stage of which, the molecular recognition of homologous (similar or identical) duplexes, remains poorly understood. Here we detect and quantify homologous recognition in protein-free ionic conditions, finding dsDNA–dsDNA recognition to be two orders-of-magnitude weaker than ssDNA–ssDNA base pairing, as explained via electrostatic helical coherence, supporting the hypothesis that purely physical recognition plays a role in homologous recombination.

Beyond canonical base pairing (1, 2), nucleic acids are known to support a diverse range of molecular interactions, from Hoogsteen hydrogen bonding (3, 4) to wobble (5, 6) and sheared (7) base pairing. These interactions underpin noncanonical structures such as triplexes, G-quadruplexes, and i-motifs, initially discovered in vitro and later found to have biological relevance (8–11). All well-characterized noncanonical interactions involve unpaired single-stranded DNA, with the nucleobases binding via hydrogen bonding and stacking. Interactions between base-paired double-stranded (ds) DNA, aside from unspecific electrostatics, are less intuitive because the information-carrying nucleobases are buried within the helical structure. However, dsDNA–dsDNA interactions are central to several biological processes (12, 13).

Among these processes is homologous recombination, through which two stretches of dsDNA sharing similar or identical nucleobase sequence come into molecular contact to initiate the exchange of genetic information (14). Homologous recombination is ubiquitous across all kingdoms of life and underpins critical processes including DNA repair, replication, chromosomal crossover, and horizontal gene transfer (15). Homologous recombination is aided by protein machinery, which orchestrates DNA resection (16), facilitates strand invasion and exchange (17), stabilizes joint molecule intermediates (18), and ensures accurate alignment and repair (19). Yet, open questions remain on the mechanism through which the initial step of homologous recombination, involving the alignment of homologous DNA stretches at molecular distances, may occur (20, 21). These have prompted the search for physical, protein-independent, mechanisms for sequence-specific interactions between dsDNA (22–28).

Experimental studies leveraging electrophoresis (29), magnetic tweezers (30) and phase separation in DNA liquid crystals (31) have shown that homologous DNA duplexes can recognize each other as intact structures, in the absence of cross-over base-pairing (32). Helical coherence theory (33, 34) has sought to rationalize these interactions as the result of sequence-specific distortions in the surface-charge distribution of DNA, which are accentuated by adsorbed counterions (35–37). Specifically, it has been hypothesized that alignment between sequence-dependent charge patterns can reduce electrostatic repulsion and, in certain regimes, generate attraction between homologous DNA stretches (33, 34). Despite accumulated evidence, however, direct experimental quantification of this, or any physical mechanism for homologous recognition, is missing. Similarly, we lack a systematic characterization of the role that physiological counterions play in modulating electrostatic interactions between dsDNA, which would be required to validate a mechanistic, quantitative theory of homology recognition.

Here we use a simple, yet highly optimized, ‘DNA tweezers’ nanosensor (38) to detect and quantify homology-dependent interactions between dsDNA, under broad ranges of ionic conditions. In the presence of physiological divalent cations, Mg^2+^ and Ca^2+^, we measure a recognition free energy of $∼-0.02k_{B}T$ (∼−0.01kcal/mol) per (pair of) base pair(s), found to be independent of cation identity and concentration, within a physiologically relevant range. We rationalize experimental evidence within the framework of electrostatic helical coherence theory, providing a description that accounts for the differential adsorption of divalent cations along the double helix, and quantitatively replicates experimental data.

These findings provide compelling, experimental and theoretical, evidence for an underinvestigated class of noncanonical interactions between intact dsDNA, independent of base pairing and the action of proteins. The recognition free energy is equivalent to ∼1% of that of canonical base pairing, and sufficient to enhance the coalignment of homologous duplexes, compared to heterologous ones, in the constrained geometry of DNA tweezers. This effect may be equally significant in cellular environments, where the high local concentration of DNA (∼10 mM base pairs in *Escherichia coli*) and relatively small free Mg^2+^ concentration (∼1 mM) (39, 40) ensures substantial Mg^2+^ adsorption coverage, which neutralizes the phosphate charges and drives duplex–duplex interactions leading to homology recognition.

## Results

### Design of FRET-Responsive DNA Tweezers.

**Fig. 1.**
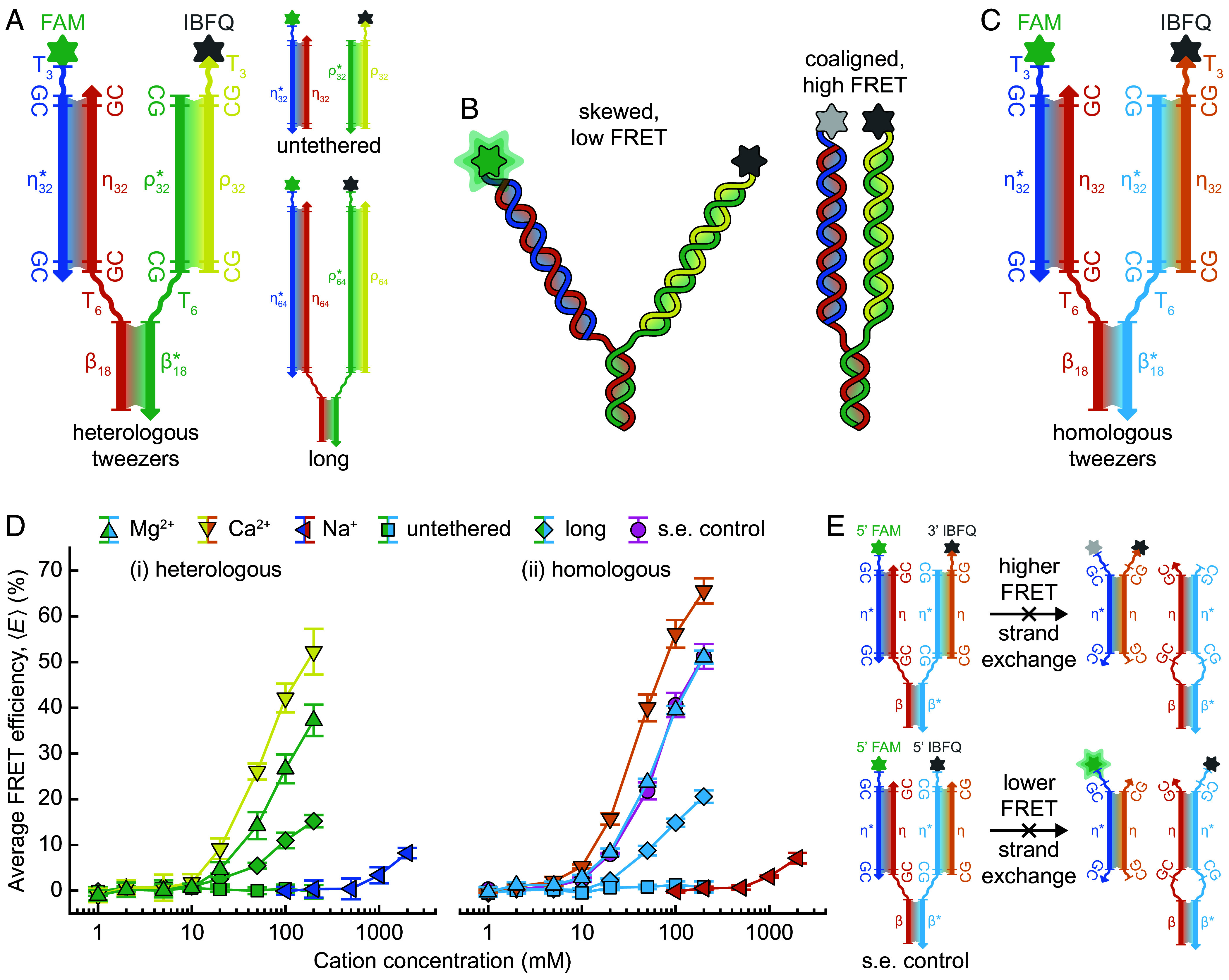
Duplex–duplex interactions are promoted by divalent cations and tethering, and enhanced by homology. (*A*) 4-strand design of heterologous DNA tweezers (*Left*). Short domain sequences are explicitly shown, Greek letters denote lengthier domains, with nucleotide lengths indicated in subscripts, and asterisks indicate reverse complementary sequences. Arrowheads indicate 3^′^ termini. FAM and IBFQ denote fluorescein and Iowa Black FQ, respectively. Also shown are untethered duplexes (*Right Top*) and long tweezers (*Right Bottom*). (*B*) Illustrations of a skewed, low-FRET, configuration (*Left*) and a coaligned, high-FRET, configuration (*Right*) of DNA tweezers. (*C*) 4-strand design of homologous tweezers. (*D*) Ensemble-averaged FRET efficiency against cation concentration for (*i*) heterologous and (*ii*) homologous tweezers (triangles) in the presence of Mg^2+^ (up-pointing), Ca^2+^ (down-pointing) and Na^+^ (*left*-pointing), and untethered duplexes (squares), long tweezers (diamonds), and strand-exchange control tweezers (circles) in the presence of Mg^2+^. Buffer, 10 mM TRIS with 100 mM NaCl and additional MgCl_2_, CaCl_2_, or NaCl; DNA construct concentration, 10 nM; data points are the mean ± SE of 3 independent repeats, each independent repeat is the mean of 4 technical repeats; lines are guides for the eye. (*E*) Illustrations of the quencher-position-dependent anomalous FRET that would arise if strand exchange occurred for homologous tweezers.

DNA tweezers, as illustrated in Fig. 1*A*, *Left*, were designed to provide control over the proximity and mutual orientation of dsDNA segments, thus enabling precise quantification of their tendency to coalign. The device consists of two 36 base-pair (bp) duplexes-of-interest (simply “duplexes” henceforth) connected through an 18 bp tethering duplex. FRET donors and acceptors, fluorescein (FAM) and Iowa Black FQ (IBFQ), respectively, are located at the free ends of duplexes, connected via T_3_ linkers to inhibit unwanted stacking interactions, such that FRET reports duplex–duplex coalignment (Fig. 1*B*). In the general, heterologous case, the central 32 bp domains of these duplexes have independent random sequences with ∼50% GC content. We denote these duplex sequences by $\eta :\eta^{*}$ and $\rho :\rho^{*}$, where η and ρ represent 32 nucleotide (nt) sequences with ∼25% of A, T, G, and C content, asterisks denote reverse complementary sequences, and colons indicate hybridization. These duplexes terminate with 2 nt GC/CG clamps which minimize end “fraying” (41, 42). Between the duplexes and the tethering duplex, $\beta :\beta^{*}$, are T_6_ linkers that are fully flexible even in the lowest ionic strength used here, having contour lengths at least three times greater than their persistence length (43). The design of homologous DNA tweezers is illustrated in Fig. 1*C*, where the central 32 base pairs of the acceptor-bearing duplex are now identical to those of the donor-bearing duplex, i.e., ρ→η. Note that the 2 nt heterologous GC/CG clamps are maintained to thermodynamically and kinetically inhibit strand exchange, as discussed in greater detail below and in *SI Appendix*, Fig. S1. The nucleobase sequences of the domains and strands used in these constructs, as determined using NUPACK (44), are given in *SI Appendix*, Table S1.

The tethered constructs provide an effective confinement of the two duplexes, whose free ends are constrained to a thin spherical shell with a radius approximately equal to the duplex length. Free movement of the duplexes about their focal point, and rotation about their connectors, is unrestricted, which is essential in enabling duplexes to find their favorable juxtaposition and mutual azimuthal orientation (34).

The ensemble-averaged FRET efficiency, ⟨E⟩, of these constructs reports the instantaneous fraction of duplexes occupying high-FRET, coaligned configurations. This observable can be calculated as $\left\langle E\right\rangle =1-I_{\mathrm{DA}}/I_{D}$, where $I_{\mathrm{DA}}$ and $I_{D}$ are donor-fluorescence intensities measured from equivalent construct solutions, one having both donor and acceptor and the second only including the donor, respectively. This strategy for estimating the FRET efficiency is independent of construct concentration, here chosen as 10 nM, provided that this is matched between donor-acceptor and donor-only solutions. A two-stage construction process was carefully designed to ensure that this requirement is fulfilled as accurately as possible (*Materials and Methods* and *SI Appendix*, Fig. S2). The careful thermodynamic design of the constructs (*SI Appendix*, Fig. S1), together with the chosen strand stoichiometries and assembly protocol (*SI Appendix*, Fig. S2), ensures a very low likelihood of donors being present in incomplete constructs (*Materials and Methods*).

### Cation-Mediated Interactions Between Heterologous and Homologous Duplexes.

Fluorimetry experiments were conducted to determine the ensemble-averaged FRET efficiency of heterologous and homologous tethered (tweezers) and untethered duplexes, in different ionic environments (Fig. 1*A* and *C*). Starting with heterologous tweezers, as shown in Fig. 1*D*(i), no FRET is observed in a standard TRIS-NaCl buffer, indicating that 100 mM monovalent Na^+^ cations do not sufficiently neutralize anionic duplexes to enable their mutual proximity for coalignment. Indeed, only at very high, ≥1 M, Na^+^ concentrations is a small FRET signal observed. The inclusion of divalent cations, however, results in more significant coalignment occurring at moderate concentrations. For magnesium, measurable FRET emerges at 20 mM Mg^2+^ and reaches ⟨E⟩=37.3(±3.4)% for 200 mM Mg^2+^. With calcium, we measure ⟨E⟩=52.3(±5.0)% for 200 mM Ca^2+^. Evidence that Ca^2+^ promotes duplex coalignment more strongly than Mg^2+^ is consistent with the view that calcium cations adsorb more readily onto dsDNA (45, 46), thereby counteracting anionic repulsion more efficiently. Verification that the FRET signal originates from “intramolecular” interactions between duplexes within the same construct, as opposed to “intermolecular” interactions between different constructs, is provided in *SI Appendix*, Fig. S3, where FRET is shown to be construct-concentration independent in the vicinity of the 10 nM concentration used here.

Fig. 1*D*(i), thus, demonstrates that moderate concentrations of divalent cations strongly promote duplex–duplex coalignment in tethered DNA constructs. Control experiments with untethered duplexes produce no FRET at any tested Mg^2+^ concentration (≤200 mM), demonstrating that coalignment is entirely dependent on duplex tethering, see Fig. 1*A*, *Top Right* and *D*(i).

To explore the effect of duplex length, tethered constructs were created with longer, 68 bp duplexes, illustrated in Fig. 1*A*, *Bottom Right*. As shown in Fig. 1*D*(i), we observe a smaller FRET response to Mg^2+^, compared to the signal recorded for the shorter, 36 bp duplexes. This finding may appear counterintuitive, as one might expect stronger attractive interactions between longer duplexes to increase the stability of coaligned, high-FRET configurations. Increasing duplex length, however, also increases their flexibility, an effect that is exacerbated by divalent cations lowering dsDNA persistence length (47). Greater duplex flexibility expands the conformation space of the nanosensor, increasing the lifetime of non-coaligned, low-FRET configurations. Theoretical modeling in *SI Appendix*, section S1 confirms that the latter entropic effect more than compensates for the stronger attraction between longer duplexes, consistent with experimental evidence.

We then proceed to examine cation-dependent duplex coalignment in homologous tweezers. Trends, reported in Fig. 1*D*(ii), are qualitatively similar to those of heterologous tweezers, but the FRET responses to divalent cations are noticeably enhanced. The onset of duplex coalignment, i.e., nonzero FRET, is observed at lower divalent-cation concentrations, 10 mM compared to 20 mM for heterologous duplexes. The average FRET efficiency also reaches higher values for homologous tweezers, 51.1(±1.4)% and 65.6(±2.8)% for 200 mM Mg^2+^ and Ca^2+^, respectively. No FRET is again observed for untethered duplexes and the response of tweezers to monovalent cations is still meager, and not enhanced relative to heterologous tweezers. Finally, the response of 68 bp tweezers is again lessened compared to 36 bp tweezers, but is greater than their heterologous equivalent. Note that, since duplex sequences $\eta :\eta^{*}$ and $\rho :\rho^{*}$, in either standard, 36- or long, 68-bp tweezers, have random nucleotide content, they will have similar, typical cation adsorption characteristics (48). Thus, for given ionic conditions, there will be no significant difference in the persistence lengths of duplexes comprising heterologous ($\eta :\eta^{*}$ and $\rho :\rho^{*}$) and homologous ($\eta :\eta^{*}$ and $\eta :\eta^{*}$) constructs. A direct comparison between homologous and heterologous systems is provided in *SI Appendix*, Fig. S4.

### Controlling Against Strand Exchange.

Evidence of a substantial homology-induced enhancement in duplex coalignment, prompted us to systematically consider, and rule out, sources of experimental artifacts. To this end, we note that homologous constructs may be able to convert to off-target complexes, in which the η and $\eta^{*}$ domains of the two duplexes undergo a strand exchange reaction, as outlined in Fig. 1*E*. For the design of homologous tweezers shown in Fig. 1*C*, should strand exchange occur, the resultant off-target complex would colocalize the FRET markers to give anomalous high FRET (Fig. 1*E*, *Top*), which would be mistaken for duplex coalignment.

Several countermeasures were adopted to prevent the formation of these off-target complexes. First, the 2 nt heterologous GC/CG clamps at both ends of the duplexes stabilize the on-target structure both thermodynamically, as these domains would be unpaired in the off-target complexes, and kinetically, as heterologous frayed ends cannot initiate strand exchange and should be minimized by GC nucleotide identity; see *SI Appendix*, Fig. S1 for further discussion and thermodynamic calculations. Second, our two-stage mixing/annealing preparation process, outlined in *SI Appendix*, Fig. S2, further kinetically inhibits the formation of off-target complexes. Since we consistently obtain ensemble-averaged FRET efficiencies within error of zero for homologous tweezers in low concentrations of divalent cations, see Fig. 1*D*(ii), strand exchange has clearly been inhibited in these conditions, a nonzero offset would be obtained if not.

Despite these countermeasures, to rule out the observed FRET enhancement for homologous constructs resulting from off-target complexes, we created homologous tweezers with the FRET acceptor (IBFQ) repositioned from the $3^{\prime }$-terminus of one strand to the $5^{\prime }$-terminus of the adjacent strand. Should strand exchange occur for this control design, the resultant off-target complexes would delocalize the FRET markers to give anomalous low FRET (Fig. 1*E*, *Bottom*), which would be mistaken for non-coaligned, skewed duplexes. Fig. 1*D*(ii) shows the FRET response of this strand-exchange control ($5^{\prime }$ IBFQ) construct to Mg^2+^—the behavior is indistinguishable from the equivalent $3^{\prime }$ IBFQ construct, confirming that strand exchange does not occur in these experiments and that the observed homology-enhanced FRET is not of anomalous origin.

### Controlling Homology via Orientation of Conserved Duplex Sequences.

Having ruled out strand-exchange artifacts as the origin of the homology-enhanced duplex–duplex interactions, we seek to strengthen the result by generalizing it to different DNA sequences. In Fig. 1, homologous and heterologous constructs have identical donor-bearing duplexes, while the acceptor-bearing duplexes were similar only in their ∼50% GC content. Because divalent cations are known to adsorb onto dsDNA with some sequence specificity, two duplexes with identical nucleobase content but different sequences may have different cation adsorption characteristics (48). To rule out such sequence-dependent effects as the origin of the differences between homologous and heterologous constructs, we take advantage of the tethering nature of the tweezers construct to control homology while conserving sequence.

**Fig. 2.**
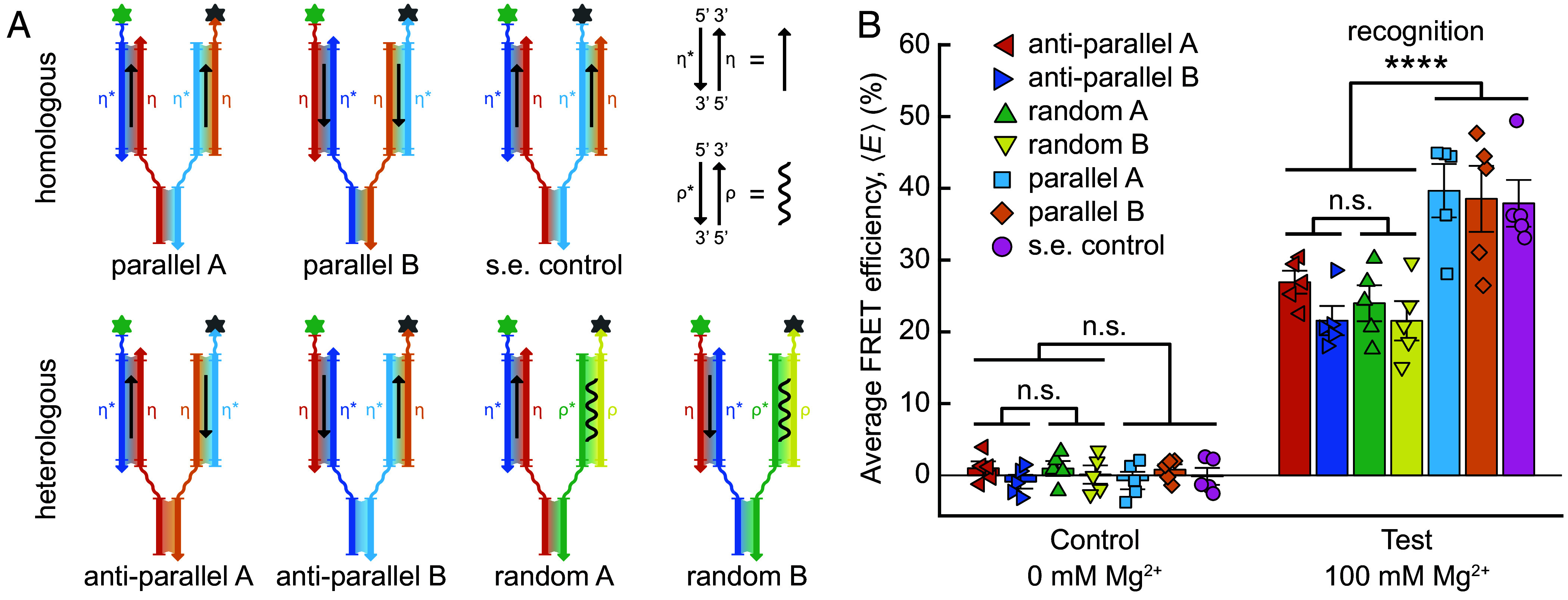
Homologous recognition is exhibited across multiple heterologous and homologous constructs. (*A*) Illustrations of homologous and heterologous constructs where arrows indicate the $5^{\prime }\to 3^{\prime }$ direction of sequence η in $\eta :\eta^{*}$ duplexes, such that [anti-]parallel arrows denote homologous [heterologous] constructs due to mutual orientation enforced by tethering. Wavy lines indicate $\rho :\rho^{*}$ duplexes in constructs that are heterologous due to mutually random duplex sequences. (*B*) Ensemble-averaged FRET efficiency in the absence (control) and presence (test) of 100 mM Mg^2+^ for the seven constructs shown in (*A*). Buffer, 10 mM TRIS with 100 mM NaCl without/with 100 mM MgCl_2_; DNA construct concentration, 10 nM; bars are the mean ± SE of 5 independent repeats (markers), each independent repeat is the mean of 4 technical repeats; unpaired *t* tests, n.s. *P* > 0.05, ****P < 0.0001 (see *SI Appendix*, Table S2 for full statistical details).

As shown in Fig. 2*A*, we tested three homologous constructs and four heterologous constructs. First, we consider the homologous construct from Fig. 1*C*, referred to here as “parallel A” due to the parallel alignment of the duplex sequences. By using different strands, both these duplexes can be reoriented to create a different homologous construct, “parallel B.” The third homologous device is the strand exchange control discussed in Fig. 1*E*, identical to the parallel A design except for the positioning of the FRET acceptor. Starting from the parallel A and B designs, inverting the orientation of only the acceptor-bearing duplex generates heterologous constructs “antiparallel A and B,” respectively, where the two duplexes have identical sequences and only differ in orientation. The final two constructs, “random A and B,” are heterologous due to sequence and are built by replacing the acceptor-bearing duplex in either A and B designs, as already shown in Fig. 1*A*.

Fig. 2*B* shows the average FRET efficiency of all seven constructs either in the absence of divalent cations or with 100 mM Mg^2+^. No FRET is observed for any construct without magnesium, as expected, while appreciable FRET is detected in all cases when magnesium is included. The average FRET efficiencies are 39.7(±3.3)% and 38.5(±4.1)% for parallel constructs and 26.9(±1.4)% and 21.6(±1.8)% for antiparallel constructs (A and B, respectively, in both cases). Clearly, parallel, homologous constructs exhibit greater FRET than their antiparallel, heterologous counterparts, ruling out homology-enhanced FRET being due to sequence-specific divalent cation adsorption. Further to this, no significant difference is observed in the Mg^2+^-induced FRET of antiparallel and random heterologous constructs, demonstrating that any sequence-specific adsorption effects are imperceptible. Finally, the overall effect of homology is found to be extremely significant (*P* < 0.0001), with homologous recognition enhancing the average FRET efficiency by 15.2(±2.2)%.

### Quantifying Homologous Recognition.

**Fig. 3.**
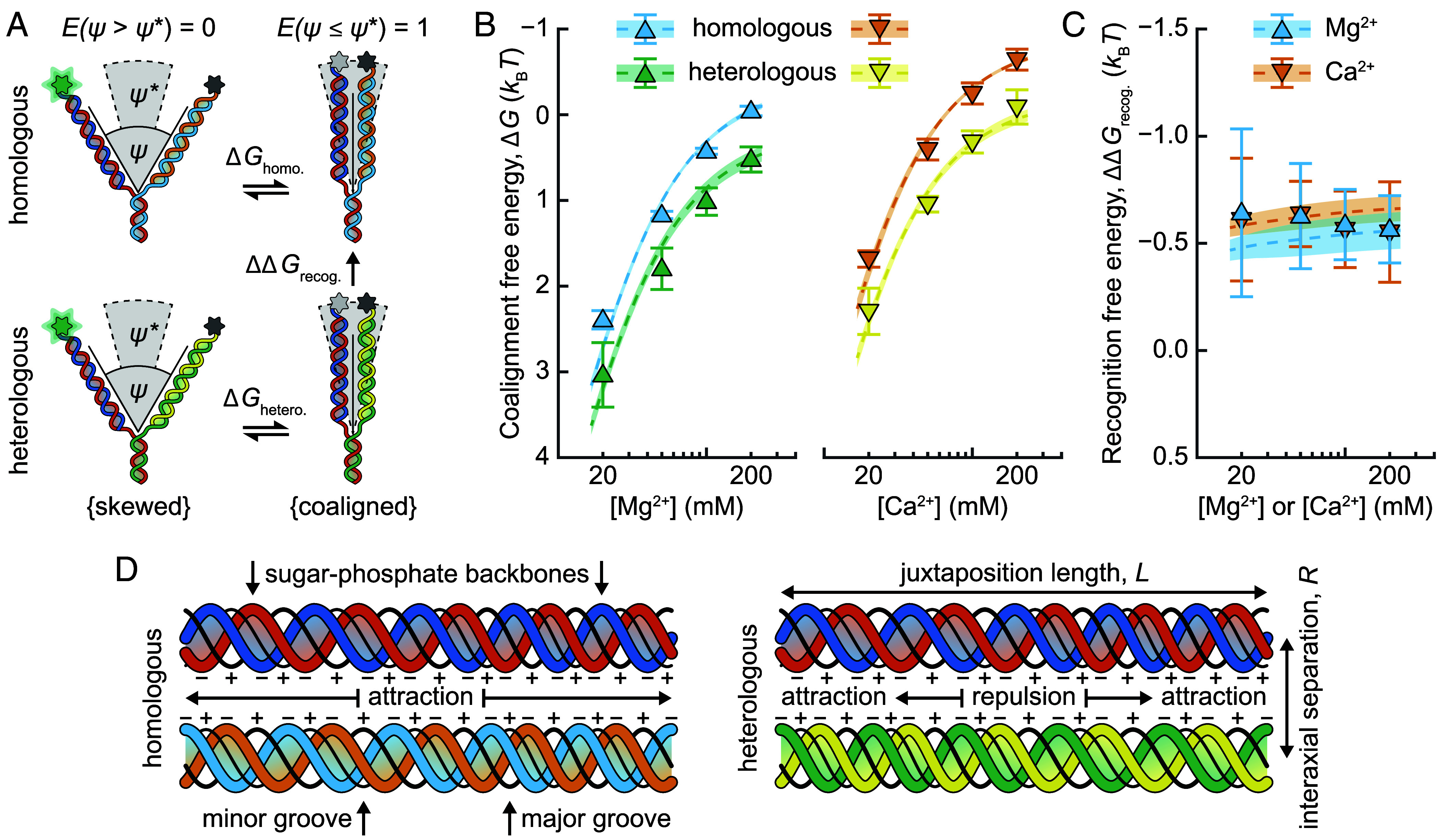
Quantification of homologous recognition, in agreement with helical coherence theory. (*A*) Illustrating skewed states and coaligned states of DNA tweezers, defined by FRET efficiencies $E(\psi >\psi^{*})=0$ and $E(\psi \leq \psi^{*})=1$, respectively, whose relative occupancies are dictated by the homology/heterology-dependent coalignment free energy, ΔG, the homologous enhancement of which is the recognition free energy, $\Delta \Delta G_{\mathrm{recog}.}$. (*B*) Coalignment free energy of homologous and heterologous tweezers against cation concentration for Mg^2+^ (*Left*) and Ca^2+^ (*Right*) cations, using data from Fig. 1*D*. Also shown are electrostatic modeling fits, Mg^2+^: $K_{d}=16.2\left(\pm 0.6\right)\;\mathrm{mM}$, $f_{2}=0.991\left(\pm 0.021\right)$, R=27.9(±0.1) Å; Ca^2+^: $K_{d}=12.0\left(\pm 0.4\right)\;\mathrm{mM}$, $f_{2}=0.999\left(\pm 0.015\right)$, R=27.7(±0.1) Å; dashed lines: best fits; shaded area: ±1σ fit confidence bounds. (*C*) Recognition free energy against divalent cation concentration, calculated from the data/fits in (*B*). (*D*) Helical coherence theory of homology recognition rests on sequence-dependent commensurate charge patterns. When two homologous dsDNA stretches interact, counterions condensed in the minor and major grooves create correlated alternating charge patterns, and the duplexes are able to rotate to allow net attraction along their lengths. For heterologous dsDNA stretches, uncorrelated sequence-dependent structural variations lead to extended regions of repulsion and narrower regions of attraction, leading to increased net repulsion.

To quantify homologous recognition beyond enhanced FRET, a simple model is applied, as illustrated in Fig. 3*A*. The model defines skewed states and coaligned states as those with low (E=0) and high (E = 1) FRET efficiencies, respectively, and assumes the relative occupancies of these states to be governed by Boltzmann statistics. Under these assumptions, the coalignment free energy, defined as the free energy difference between coaligned states and skewed states, is given by

[1] $$\begin{array}{c} \Delta G=-k_{B}T\ln\left(\frac{\left\langle E\right\rangle }{1-\langle E\rangle }\right), \end{array}$$

where $k_{B}T$ is thermal energy (see *SI Appendix*, section S2 for derivation). The homologous recognition free energy is thus simply defined as the difference between the coalignment free energies of homologous and heterologous constructs in identical conditions, namely,

[2] $$\begin{array}{c} \Delta \Delta G_{\mathrm{recog}.}=\Delta G_{\mathrm{homo}.}-\Delta G_{\mathrm{hetero}.}. \end{array}$$

Applying this model to data from Fig. 2*B*, where ${\left\langle E\right\rangle }_{\mathrm{homo}.}=38.7\left(\pm 1.9\right)\%$ and ${\left\langle E\right\rangle }_{\mathrm{hetero}.}=23.5\left(\pm 1.1\right)\%$ for $[{\mathrm{Mg}}^{2+}]=100\;\mathrm{mM}$, gives $\Delta \Delta G_{\mathrm{recog}.}=-0.721\left(\pm 0.102\right)\;k_{B}T$. Since these homologous constructs possess 32 pairs of homologous base-pairs, $\Delta \Delta G_{\mathrm{recog}.}\approx -0.02\;k_{B}T$ per (pair of) base pair(s). This recognition interaction is approximately two orders of magnitude smaller than the average base-pairing interactions responsible for duplex stability, $\Delta G_{\mathrm{BP}}\approx -2.5k_{B}T$ per base pair in typical ionic conditions (49). The effect of relaxing the step-function approximation of the FRET efficiency used in this analysis is examined in *SI Appendix*, section S8. This more complete treatment is found to lead to only a minor correction to the inferred recognition free energies, and does not alter the conclusions.

To explore how divalent cation identity (Mg^2+^ or Ca^2+^) and concentration affect homologous recognition, Eqs. **1** and **2** are applied to the ensemble-averaged FRET efficiencies of homologous (parallel A) and heterologous (random A) tweezers from Fig. 1*D*, considering the concentration range over which ⟨E⟩ is nonzero for all construct/cation combinations (divalent cation concentration ≥20 mM). This produces the concentration-dependent coalignment and recognition free energies shown in Fig. 3 *B* and *C*, respectively. Remarkably, all recognition free energies agree within error, suggesting that homologous recognition is independent of cation identity and concentration, over the tested range spanning an order of magnitude. It is clear that reasonably high concentrations, $∼10^{2}\;\mathrm{mM}$, of divalent cations are required for substantial (∼50%) FRET, but moderate concentrations, $∼10^{1}\;\mathrm{mM}$, are sufficient for recognition. In other words, while elevated levels of divalent cations enable more-frequent duplex–duplex interactions, they do not significantly affect the interaction differential between homologous and heterologous duplexes.

### Mechanistic Insights with Helical Coherence Theory.

To elucidate the microscopic origin of homologous recognition, we seek to compare our experimental measurements with predictions from helical coherence theory (HCT). HCT is based on the idea that dsDNA exhibits small, sequence-dependent deviations from an ideal double-helical structure due to variations in twist angle and base-pair rise depending on the identities of neighboring base pairs (33, 35–37). Homologous stretches of dsDNA have identical, or very similar, sequences, leading to correlated charge patterns produced by the anionic phosphate groups of the backbone and adsorbed counterions. When two such homologous duplexes are coaligned in a parallel configuration, rotation around their longitudinal axes can register positive charges on one duplex to negative charges on the other, as illustrated in Fig. 3*D*. This registration reduces electrostatic repulsion, and may lead to short-range attraction, depending on the coverage and spatial distribution of the counterions (34). In contrast, heterologous duplexes accumulate mismatches along their length, preventing such optimal charge registration. The characteristic length over which structural correlations between nonhomologous sequences are lost is known as the helical coherence length, $\lambda_{c}$, which has been theoretically and experimentally estimated as $\lambda_{c}\approx 11$ nm (50, 51). Note that, in HCT, homologous duplexes aligned antiparallel interact in an equivalent way to heterologous duplexes (52).

Remarkably, as shown in *SI Appendix*, Fig. S5, all experimental estimates of $\Delta \Delta G_{\mathrm{recog}.}$ from Figs. 2*B* and 3*C* fall squarely within the range of affinities recently predicted by HCT (34), without any parameter fitting. Encouraged by this striking agreement, we proceed to derive, within the framework of HCT, an expression for the duplex coalignment free energy, ΔG, consistent with the geometry of our tweezers constructs, which can be used to fit experimental trends. ΔG must be expressed in terms of a duplex–duplex interaction potential, U(ψ), where *ψ* is the duplex–duplex skew angle. To this end, we approximate the FRET efficiency as a step function, with $E(\psi \leq \psi^{*})=1$ and $E(\psi >\psi^{*})=0$, where $\psi^{*}=2\arcsin\left(R_{F}/2L\right)$ is the skew angle at which the donor-acceptor separation equals the Förster radius, $R_{F}$ (*Materials and Methods* and *SI Appendix*, section S3), and L=12.2 nm is the length of 36 bp duplexes. Under the additional assumption that U(ψ) is effectively constant for $\psi \leq \psi^{*}$, we can write

[3] $$\begin{array}{c} \Delta G=U^{\|}+k_{B}T\ln\left[\Omega \left(U^{∠}\right)\right], \end{array}$$

where $U^{\|}$ is the interaction potential of coaligned duplexes and

[4] $$\begin{array}{c} \Omega \left(U^{∠}\right)=\frac{1}{1-\cos(\psi^{*})}\int_{\psi^{*}}^{\pi }d\psi \;\sin\left(\psi \right)e^{-U^{∠}\left(\psi \right)/k_{B}T}, \end{array}$$

where $U^{∠}\left(\psi \right)$ is the interaction potential of skewed duplexes (see *SI Appendix*, section S4 for derivation).

HCT can then be considered to determine expressions for both $U^{\|}$ and $U^{∠}\left(\psi \right)$. For the coaligned potential, HCT gives (34)

[5] $$\begin{array}{c} U^{\|}\left(R,L;\theta ,f_{1},f_{2}\right)=L\sum_{n=0}^{\infty }a_{n}^{\|}\left(R;\theta ,f_{1},f_{2}\right)\nu_{n}\left(R,L\right), \end{array}$$

where *R* is interaxial separation, *L* is the length of DNA juxtaposition, *θ* is the portion of the phosphate charge neutralized by adsorbed cations (“overall charge compensation” henceforth), and $f_{1}$ and $f_{2}$ are the fractions of adsorbed cations in the minor and major grooves, respectively, with $f_{1}+f_{2}=1$. The functions $a_{n}^{\|}$ and $\nu_{n}$ are the parallel helical charge harmonics and recognition coefficients, respectively; full expressions are presented in *SI Appendix*, section S5.

To compute $U^{∠}$, we consider $\psi >\psi^{*}$. For these skew angles, the lengths of dsDNA stretches that are sufficiently close to interact, estimated as $\lambda_{D}/\left|\sin\psi \right|$, where $\lambda_{D}$ is the Debye length, become much shorter than the helical coherence length, $\lambda_{c}$. Therefore, in the skewed regime, homologous and heterologous duplexes can be considered indistinguishable. Additionally, because electrostatic interactions from regions of the duplexes distant from their connection point become negligible, $U^{∠}$ may be approximated as the interaction between two semi-infinitely extended, ideal double helices, namely

[6] $$\begin{array}{c} U^{∠}\left(R,\psi ;\theta ,f_{1},f_{2}\right)=\frac{1}{\left|\sin\psi \right|}\sum_{n=-\infty }^{\infty }\sum_{m=-\infty }^{\infty }a_{\mathrm{nm}}^{∠}\left(R,\psi ;\theta ,f_{1},f_{2}\right), \end{array}$$

where $a_{\mathrm{nm}}^{∠}$ are the skewed helical charge harmonics; their full expression is presented in *SI Appendix*, section S6.

In computing the parallel and skewed helical harmonics, we use a nonlocal continuum electrostatic description of the electrolyte, coupling the spatially dispersive dielectric response of water to Debye–Hückel ionic screening (see *SI Appendix*, section S5 for justification), thereby capturing how solvent polarization and ions modify the electric field around dsDNA (53) and mediate dsDNA–dsDNA interactions (34, 54). Finally, the overall charge compensation, *θ*, is modeled with a Langmuir isotherm,

[7] $$\begin{array}{c} \theta \left(c\right)=\frac{c}{K_{d}+c}, \end{array}$$

where *c* is the bulk concentration of divalent cations and $K_{d}$ is the dissociation constant for cation adsorption onto dsDNA.

Within this framework, we fit the experimental data in Fig. 3*B* to Eq. **3** using $U^{\|}$ and $U^{∠}\left(\psi \right)$ from Eqs. **5** and **6**, respectively, with only three fitting parameters per cation type: i) the cation dissociation constant, $K_{d}$; ii) the fraction of major-groove-adsorbed cations, $f_{2}$ (with $f_{1}=1-f_{2}$); and, iii) the interaxial separation, *R*. For each cation type, fitting is conducted simultaneously on homologous and heterologous data, with $U^{\|}$ calculated analytically and $\Omega \left(U^{∠}\right)$ numerically (see *SI Appendix*, section S7 for details). As shown in Fig. 3*B*, this framework produces excellent fits. Remarkably, the optimized cation dissociation constants, $K_{d}=16.2\left(\pm 0.6\right)\;\mathrm{mM}$ and $K_{d}=12.0\left(\pm 0.4\right)\;\mathrm{mM}$ for Mg^2+^ and Ca^2+^, respectively, correlate well with the onset of FRET observed Fig. 1*D*. Notably, the associated major-groove adsorption fractions, $f_{2}=0.991\left(\pm 0.021\right)$ and $f_{2}=0.999\left(\pm 0.015\right)$ for Mg^2+^ and Ca^2+^, respectively, suggests a dominance of major-groove binding. Previous experimental and computational studies have shown that divalent cations can bind in both the major and minor grooves, although reported distributions vary depending on the experimental approach, ionic environment, and DNA sequence content (55–58). Theoretical work further suggests that closer duplex spacing can drive ion redistribution toward the major groove (59). Our experiments, involving relatively small interaxial separations in mixed monovalent/divalent ionic environments, fall within the regime where major-groove enrichment is expected. Within this context, these $f_{2}$ values should be interpreted as effective, condition-specific parameters that reflect the dominant adsorption behavior in our experiments, rather than implying cations exclusively occupy one groove. The optimized interaxial separations, R=27.9(±0.1) Å and R=27.7(±0.1) Å for Mg^2+^ and Ca^2+^, respectively, correspond well to surface–surface separations similar to, or slightly smaller than, the Debye length, as required for the electrostatic interactions of fine patterns to remain significant compared to repulsion generated by the net negative charge. Finally, Fig. 3*C* shows that both the magnitude and concentration-insensitivity of homologous recognition are well recapitulated by HCT. As discussed in *SI Appendix*, section S8, relaxing the step-function approximation for the FRET efficiency in the electrostatic model leads only to a modest quantitative correction and does not materially alter the resulting free-energy estimates.

We note that, while finite experimental sensitivity prevented the quantification of coalignment and recognition free energies for concentrations of divalent cations below 20 mM (Fig. 3 *B* and *C*), theoretical fits can be extrapolated to lower cation concentrations (1 mM, see *SI Appendix*, Fig. S6). This exercise is useful to explore regimes that may be relevant to intracellular environments, reported to have free [Mg^2+^] (the most abundant divalent cation) of order ∼1 mM. We predict that, while the coalignment free energy expectedly drops substantially with decreasing [Mg^2+^] (*SI Appendix*, Fig. S6*A*), the recognition free energy only displays a marginal decrease (*SI Appendix*, Fig. S6*B*). This suggests that homologous recognition should remain substantial, provided that suitable mechanisms are in place to overcome charge repulsion between duplexes. Crowding, which is prominent in cellular environments, could be one such mechanism, consistent with experimental observations (31). We further note that, while free [Mg^2+^] is low in cellular environments, total [Mg^2+^] is considerably more so, ∼20 mM (60), with the majority bound to nucleic acids and other cellular components. It is thus expected that the coverage of cation adsorption sites should remain high, similar to those predicted in our experiments with higher free [Mg^2+^].

Ionic conditions influence homologous recognition via electrostatic effects, as demonstrated in this work, but also, potentially, via modulating dsDNA’s mechanical properties. Changes in bending and torsional persistence lengths, due to cation adsorption, can alter the manner in which long duplexes accommodate sequence-dependent helical mismatches. In particular, torsional flexibility enables partial adjustment of accumulated phase mismatch, moderately reducing the recognition energy at the cost of elastic deformation (for a review, see ref. 61). Thus, for long duplexes, where elastic degrees of freedom play an important role, ionic modulation of dsDNA rigidity could provide an additional pathway by which cation identity and concentration affect recognition. The length of the short, 36 bp duplexes used in our experimental determinations of recognition free energy are well below the bending and torsional persistence lengths of dsDNA; they are essentially rigid. Therefore, cation-dependent variations in elasticity will not manifest themselves in these inferences.

## Discussion

We demonstrate that, in conjunction with refined measurements protocols, FRET-based “DNA tweezers” inspired by previously introduced DNA nanosensors (38, 62–64), can detect and accurately quantify subtle sequence- and cation-dependent interactions between dsDNA duplexes. We implemented this method to demonstrate that physiological divalent (Mg^2+^ and Ca^2+^) cations are substantially more effective in promoting coalignment between DNA duplexes compared to monovalent (Na^+^) cations.

By controlling duplex sequence and mutual orientation, we demonstrate that the tendency for coalignment is strongest for homologous duplexes, which share identical nucleobase sequences, compared to heterologous duplexes. The observation is extremely statistically significant (*P* < 0.0001 for 100 mM Mg^2+^), and robust against several controls conducted with different DNA sequences. These include experiments in which heterologous constructs have been generated by inverting the directionality of one duplex in a homologous pair, confirming that the enhanced affinity is the result of sequence homology, rather than arising from other forms of sequence-dependent duplex–duplex interactions. In addition, dedicated controls rule out undesired side reactions mediated by base pairing.

Accurate measurements allow us to estimate a homology-induced recognition free energy of $∼-0.02\;k_{B}T$ per base pair, which is independent of the identity (Mg^2+^ or Ca^2+^) and concentration, within the tested range, of divalent cations. This contribution is equivalent to ∼1% of typical base-pairing free energies and amounts to $∼-3\;k_{B}T$ over the scale of the dsDNA persistence length (∼150 bp or ∼50 nm). Our experiments demonstrate that this level of interaction is sufficient to promote alignment between homologous sequences at the high local DNA concentrations found in cells. Substantial Mg^2+^ adsorption is expected at ∼1 mM free Mg^2+^ (39, 40), as the concentration of DNA adsorption sites (∼10 mM base pairs) is comparable to the total cellular Mg^2+^ concentration. The recognition effect reported here, with closely related phenomena observed in earlier in vitro studies (29–31, 65), is therefore likely relevant in DNA-rich cellular environments.

Two key conclusions follow. First, the modest interaction strength favors dynamic association-dissociation of homologous duplexes over irreversible binding. Second, the recognition free energy is largely insensitive to divalent cation concentration across the range tested (Fig. 3*C*).

To substantiate the physical mechanism behind these conclusions, we proposed an electrostatic model within the framework of helical coherence theory that accounts for the thermodynamics of counterion-mediated duplex–duplex interactions. The model interprets homology recognition as originating from sequence-dependent distortions in the double helix and, consequently, in the charge patterns on the surface of dsDNA. These translate into enhanced affinity between homologous duplexes compared to the heterologous case, which our theoretical framework can predict given a small set of physically interpretable parameters. We fit these predictions to experimental data for both Mg^2+^ and Ca^2+^, using only three free parameters: the interaxial duplex–duplex separation, *R*, the fraction of divalent ions adsorbed in the major groove, $f_{2}$, and the cation-adsorption dissociation constant, $K_{d}$. For both Mg^2+^ and Ca^2+^, the optimized values of all parameters align well with intuition and literature: $K_{d}$ coincides with the onset of duplex coalignment in experiments, *R* corresponds to a distance between DNA surfaces similar to the Debye length, and $f_{2}\approx 1$ suggests that divalent cations predominantly occupy the major groove under our experimental conditions. After parameter optimization, the model quantitatively reproduces the measured dependencies on salt concentration and homology-specific interaction energies, strongly supporting this physical mechanism for homology recognition.

Taken together, these results provide conclusive insights into the magnitude and physical mechanism underpinning protein-free homology recognition in double-stranded DNA, which may play a role in ubiquitous and highly conserved biological pathways for homologous recombination.

## Materials and Methods

The nucleotide sequences of DNA strands used in experiments are shown in *SI Appendix*, Table S1. The determination of these sequences was aided by the NUPACK design tool (44) in order to maximize the formation of the on-target structure (*SI Appendix*, Fig. S1) and minimize secondary structure in the β and $\beta^{*}$ domains (such that $\beta :\beta^{*}$ hybridization is uninhibited at room temperature). All DNA strands were purchased from Integrated DNA Technologies (IDT), with high-performance liquid chromatography purification performed on oligonucleotides of length >40 nt or with terminal modifications. FRET-donor strands were modified with 6-carboxyfluorescein at the 5’ end, and FRET-acceptor strands were modified with Iowa Black FQ at either the $3^{\prime }$ or $5^{\prime }$ end. Both modifications were attached to terminal phosphate groups by phosphoramidite chemistry, via a 6-carbon linker in the case of fluorescein. DNA was shipped lyophilized and reconstituted to 100 μM in 10 mM TRIS (Sigma Aldrich) with 100 mM sodium chloride (Sigma Aldrich); this pH 8.0 buffer was used throughout all experiments. Precise concentrations of these ssDNA stock solutions were evaluated from measurements of 260 nm absorbance using a NanoDrop One microvolume UV-vis spectrophotometer (Thermo Scientific) in combination with sequence-specific extinction coefficients (provided by IDT). IDT provided the Förster radius of $R_{\text{F}}=5.79$ nm for FRET between fluorescein and Iowa Black FQ, as determined in pH 8.0 tris-EDTA.

The construction of DNA tweezers was achieved in a two-stage process, described here and illustrated in *SI Appendix*, Fig. S2 with respect to 36 bp homologous tweezers. First, donor-bearing, acceptor-bearing, and “blank” duplexes were annealed separately. For donor-bearing duplexes, strands *D* and *X* were mixed to give 100μl preannealed solutions with [D]=2.00 μM and [X]=2.52 μM; for acceptor-bearing and blank duplexes, strands *Y* and *A* or *B* were mixed to give 100μl preannealed solutions with [Y]=3.17 μM and [A] or [B]=4.00 μM, respectively. Thermal annealing was performed using a C1000 Touch thermal cycler (Bio-Rad); solutions were heated to and held at 95 ^°^C for 5 min, to ensure complete melting of any secondary structure, then cooled at a rate of 1 ^°^C every 3 min until 25 ^°^C was reached. LoBind tubes (Eppendorf) were used for the annealing process (and all proceeding preparatory steps) to minimize sample loss due to physisorption to plasticware surfaces, thereby, minimizing concentration uncertainty. Following annealing, equal volumes of solutions containing donor-bearing and acceptor-bearing or blank duplexes were mixed at room temperature and left for 1 h, during which time the formation of donor-acceptor or donor-only constructs, DXYA and DXYB respectively, occurs due to $\beta :\beta^{*}$ hybridization. To further minimize the physisorption of DNA-of-interest, particularly when diluted to the measurement concentration (typically 10 nM), all postannealed solutions contained 1μM polythymine ($T_{20}$) to sacrificially physisorb, but not to interact with the DNA-of-interest. Note that this two-stage mixing/annealing process is necessary for the preparation of homologous tweezers in order to prevent formation of the metastable, off-target complexes DA/DB and XY, which may form in a one-stage process.

Fluorescence assays were performed in Sterilin Clear Microtiter flat-bottomed 96-well plates (Thermo Scientific) where 20 μl 5× (typically 50 nM) DNA solution was added to 80 μl 1.25× cation solution to give 100 μl total volume per well (cation solutions were prepared by the addition of magnesium or calcium chloride (both Sigma Aldrich) to buffer). Prior to measurement, samples were incubated at room temperature for 3 h to ensure equilibration of cation adsorption. For these assays, fluorescent intensity was monitored with a CLARIOstar Plus microplate reader (BMG Labtech) operating with excitation and emission wavelengths suited to fluorescein, 483/14 nm and 530/30 nm, respectively. For a given condition, the emission intensities of 4 technical repeats of donor-acceptor constructs, donor-only constructs, and buffer (no DNA) were measured, the latter providing a characterization of background fluorescence. These technical repeats were averaged and the mean background intensity was subtracted from the mean construct intensities to give the background-subtracted mean donor-acceptor and donor-only intensities, $I_{\text{DA}}$ and $I_{\text{D}}$, respectively. Note that, since divalent cations are known to quench fluorescent emission, the emission intensity of donor-only constructs decreased significantly with increasing divalent cation concentration and, thus, needed to be measured in the same conditions as donor-acceptor constructs (the emission intensity of buffer did not vary in any significant way with cation concentration, as expected). The ensemble-average FRET efficiency was calculated via $\left\langle E\right\rangle =1-I_{\text{DA}}/I_{\text{D}}.$ Final values are given as the mean ± SE of values from 3 independently repeated experiments (thus, 36 emission intensity measurements in total combine for each final value). This determination of average FRET efficiency is construct-concentration independent provided that: i) donor-acceptor and donor-only constructs are the same concentration; and ii) FRET occurs intramolecularly (between markers on the same construct), not intermolecularly (between markers on different constructs). On this first point, mixing/annealing constructs in the manner described above (as opposed to mixing/annealing all four strands at once) results in the donor concentration of donor-acceptor- and donor-only-construct solutions being as similar as possible. On the second point, the use of strand excesses ensures that all donor-bearing strands become incorporated into complete constructs, e.g., for 36 bp homologous tweezers, the ∼26% excesses of [X] to [D], [Y] to [X], and [A] to [Y] cumulate to a 100% excess of [A] to [D]. This ensures that all donors in donor-acceptor constructs can undergo intramolecular FRET, meaning that measurement of donor emission alone reliably reports on average FRET efficiency (which would be underreported if donors were present in incomplete, “acceptor-less” donor-acceptor constructs). Finally, *SI Appendix*, Fig. S3 confirms that FRET is intramolecular in nature for constructs in solution at 10 nM, i.e., originating from interactions between duplexes within the same construct, as opposed to interactions between different constructs. Note that, using an emissive, instead of a nonemissive (quencher), acceptor would have allowed FRET efficiency to be determined from increased acceptor emission. This method, however, is typically less accurate (66) than the method we used (decreased donor emission) and requires absorbance measurements and precisely known donor:acceptor stoichiometries.

## Supplementary Material

Appendix 01 (PDF)

## Data Availability

All data and analysis codes are available in an online repository (67). All study data are included in the article and/or *SI Appendix*.
