# Supplementary material for: Direct evidence and quantification of homologous recognition between DNA duplexes
Source: Proc Natl Acad Sci U S A. 2026 Jun 4;123(23):e2530949123. doi: 10.1073/pnas.2530949123 (PMC13250564; doi:10.1073/pnas.2530949123)
Supplement: Supplementary file 1 — Appendix 01 (PDF) [file pnas.2530949123.sapp.pdf]

Supporting Information for

Direct evidence and quantification of homologous recognition between DNA duplexes

Andrew Stannard, Ehud Haimov, Jonathan G. Hedley, Yaxuan Xiao, Marco Di Antonio, Gleb Oshanin, Claudia Danilowicz, Mara Prentiss, Lorenzo Di Michele, Alexei A. Kornyshev

Corresponding Authors:

Lorenzo Di Michele
E-mail: ld389@cam.ac.uk

Alexei A. Kornyshev
E-mail: a.kornyshev@imperial.ac.uk

This PDF file includes:

Supporting text
Figs. S1 to S10
Tables S1 to S2
SI References

Domain	Sequence ($5' \rightarrow 3'$)
β_{18}	GCGACATAGATACCAGGC
η_{32}	ATACGTGTTACAGTTACTGAGCACTGCTGAAA
ρ_{32}	CGACTTTGTCATCAAGCATGTAGCGTCCGACG
η_{64}	AGAGTCACGAGTCCAGTCATAGGGTCCGAGGTCCAGTCACAGTCCTTAGTTCAT... CAGTTCAGTA
ρ_{64}	ATGAACCTAGCCCTACTGAACTTGACGATACTGACTGATACTGACTGAACTTGA... ACCCTGAACC

Strand (nt)	Sequence ($5' \rightarrow 3'$)	Strand (nt)	Sequence ($5' \rightarrow 3'$)
A (39)	CG η_{32} CG T_3 IBFQ	short X (36)	GC η_{32} GC
A' (63)	IBFQ T_3 CG η^*_{32} CG T_6 β^*_{18}	short Y (36)	CG η^*_{32} CG
A_F (39)	CG η^*_{32} CG T_3 IBFQ	short Y_R (36)	CG ρ^*_{32} CG
A_R (39)	CG ρ_{32} CG T_3 IBFQ	long A' (95)	IBFQ T_3 CG η^*_{64} CG T_6 β^*_{18}
B (39)	CG η_{32} CG T_3	long A'_R (95)	IBFQ T_3 CG ρ^*_{64} CG T_6 β^*_{18}
B' (63)	T_3 CG η^*_{32} CG T_6 β^*_{18}	long B' (95)	T_3 CG η^*_{64} CG T_6 β^*_{18}
B_F (39)	CG η^*_{32} CG T_3	long B'_R (95)	T_3 CG ρ^*_{64} CG T_6 β^*_{18}
B_R (39)	CG ρ_{32} CG T_3	long D (71)	FAM T_3 GC η^*_{64} GC
D (39)	FAM T_3 GC η^*_{32} GC	long X (92)	β_{18} T_6 GC η_{64} GC
D_F (39)	FAM T_3 GC η_{32} GC	long Y' (68)	CG η_{64} CG
X (60)	β_{18} T_6 GC η_{32} GC	long Y'_R (68)	CG ρ_{64} CG
X_F (60)	β_{18} T_6 GC η^*_{32} GC		
Y (60)	CG η^*_{32} CG T_6 β^*_{18}		
Y' (36)	CG η_{32} CG		
Y_F (60)	CG η_{32} CG T_6 β^*_{18}		
Y_R (60)	CG ρ^*_{32} CG T_6 β^*_{18}		

Construct type	Strands (donor-acceptor/donor-only)
heterologous tethered (random A)	D, X, Y_R, A_R/B_R
heterologous untethered	D, short X, short Y_R, A_R/B_R
heterologous long tethered	long D, long X, long A'_R/long B'_R, Y'_R
homologous tethered (parallel A)	D, X, Y, A/B
homologous untethered	D, short X, short Y, A/B
homologous long tethered	long D, long X, long A'/long B', Y'
strand-exchange control tethered	D, X, A'/B', Y'
homologous tethered (parallel B)	D_F, X_F, Y_F, A_F/B_F
heterologous tethered (anti-parallel A)	D, X, Y_F, A_F/B_F
heterologous tethered (anti-parallel B)	D_F, X_F, Y, A/B
heterologous tethered (random B)	D_F, X_F, Y_R, A_R/B_R

Table S1. Nucleotide sequences of domains, domain sequences of strands, and strand compositions of constructs. FAM and IBFQ refer to 6-carboxyfluorescein and Iowa Black FQ, respectively. Asterisks (*) refer to reverse complementary sequences. Strands indicated by primes (′) are utilized in configurations with $5'$ IBFQ.

$[\mathbf{Mg^{2+}}]$ (mM)	Construct type	$\langle E \rangle$ (%) mean $\pm$ s.e., n	Unpaired t-test p-value
0	anti-parallel	0.1 ± 0.7, 10	0.53 (n.s.)
	random	0.7 ± 0.7, 10	
	heterologous	0.3 ± 0.5, 20	0.66 (n.s.)
	homologous	0.0 ± 0.6, 15	
100	anti-parallel	24.2 ± 1.4, 10	0.41 (n.s.)
	random	22.3 ± 1.8, 10	
	heterologous	23.5 ± 1.1, 20	< 0.0001 (****)
	homologous	38.7 ± 1.9, 15	

Table S2. Details of the statistical analysis in Figure 2B. In the absence of Mg^{2+} cations, no significant difference between anti-parallel and random heterologous constructs, nor between heterologous and homologous constructs, is observed. In the presence of Mg^{2+} cations, no significant difference between anti-parallel and random heterologous constructs is observed, but the effect of homology is extremely significant.

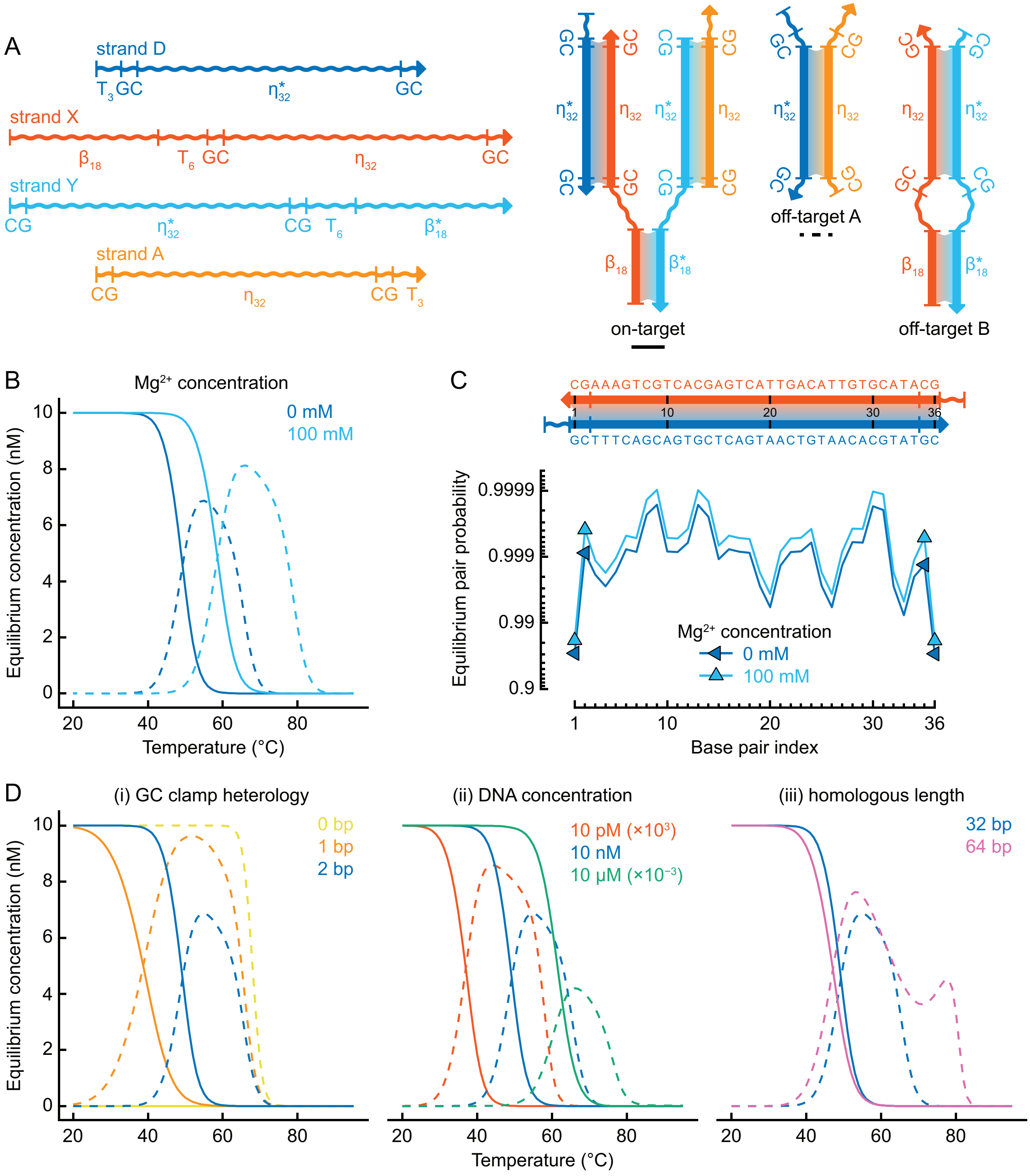

Andrew Stannard, Ehud Haimov, Jonathan G. Hedley, Yaxuan Xiao, Marco Di Antonio, Gleb Oshanin, Claudia Danilowicz, Mara Prentiss, Lorenzo Di Michele, Alexei A. Kornyshev

Fig. S1. Strand exchange does not occur in homologous tweezers due to thermodynamic and kinetic inhibition ensured by heterologous GC clamps. $\mathbf{(A)}$ Strands D (without FAM), X, Y, and A (without IBFQ) are used for NUPACK calculations of the on-target homologous complex and off-target strand-exchange complexes. Any thermodynamic drive for strand exchange will be dictated by the temperature-dependent balance between the free energy (entropic) gain of an extra complex and the free energy cost of losing 8 GC base pairs. $\mathbf{(B)}$ Equilibrium concentration against temperature for on-target (solid lines) and off-target A complexes (dashed lines), as calculated by NUPACK using 100 mM Na^{+} without (blue) and with (cyan) 100 mM Mg^{2+} for $[\mathrm{D}] = 10.0$ nM, $[\mathrm{X}] = 12.6$ nM, $[\mathrm{Y}] = 15.9$ nM, and $[\mathrm{A}] = 20.0$ nM (strand excesses are explained in Materials and Methods). With respect to FRET (including potential anomalous FRET), only the on-target and off-target A complexes need considering, see Figure 1E. The off-target B complex does not affect FRET and, thus, its temperature-dependent concentration can be ignored. At room temperature (20 $^\circ$C), the on-target complex is the only FRET-relevant complex present. As temperature increases, however, the off-target A complex becomes prevalent (before melting at high temperature). In the absence of magnesium, the temperature at which on-target and off-target A complexes have equal concentrations, i.e., the temperature characterising thermal stability with respect to strand exchange, is $T_{\mathrm{SE}} = 49\ ^\circ\mathrm{C}$. The effect of magnesium is to further stabilise the on-target complex, raising the strand-exchange temperature to $T_{\mathrm{SE}} = 59\ ^\circ\mathrm{C}$. This is evidence that strand exchange is thermodynamically-inhibited at the experimental conditions. $\mathbf{(C)}$ Equilibrium pair probability against base pair index for the donor-bearing duplex of homologous tweezers, as calculated by NUPACK using 100 mM Na^{+} without (blue, down-pointing triangles) and with (cyan, up-pointing triangles) 100 mM Mg^{2+}; base pairs of the heterologous GC clamps are highlighted by markers. Compared to base pairs in the bulk of the duplex, it is clear that only the outermost (1 and 36) are compromised in any way. Since strand exchange is most likely initiated where pair probability is lowest, i.e., at duplex termini, here it is kinetically inhibited since these base pairs are heterologous with respect to those of the acceptor-bearing duplex. The effect of Mg^{2+} is to increase equilibrium pairing and, thus, duplex stability. $\mathbf{(D)}$ Further plots of equilibrium concentration against temperature for on-target (solid lines) and off-target A (dashed lines) complexes, for 100 mM Na^{+}, discussed with respect to 10 nM homologous tweezers with 32 bp homology and 2 bp GC clamp heterology (blue). (i) Reducing GC clamp heterology to 1 bp (orange) reduces thermal stability to $T_{\mathrm{SE}} = 39\ ^\circ\mathrm{C}$, and room temperature approximately demarcates the onset of strand exchange. Completely removing GC clamp heterology (yellow) results in no tweezers structures being thermodynamically favourable at any temperature. Note that in both these cases 2 bp GC clamps are still present, it is the extent of their heterology is varied. (ii) Increasing the DNA tweezers concentration to 10 μM (green) increases the thermal stability to $T_{\mathrm{SE}} = 63\ ^\circ\mathrm{C}$. Decreasing the DNA tweezers concentration to 10 pM (red) decreases the thermal stability to $T_{\mathrm{SE}} = 37\ ^\circ\mathrm{C}$, and room temperature is, approximately, the limit of stability. (iii) Increasing the homologous length to 64 bp (magenta) slightly decreases the thermal stability to $T_{\mathrm{SE}} = 47\ ^\circ\mathrm{C}$.

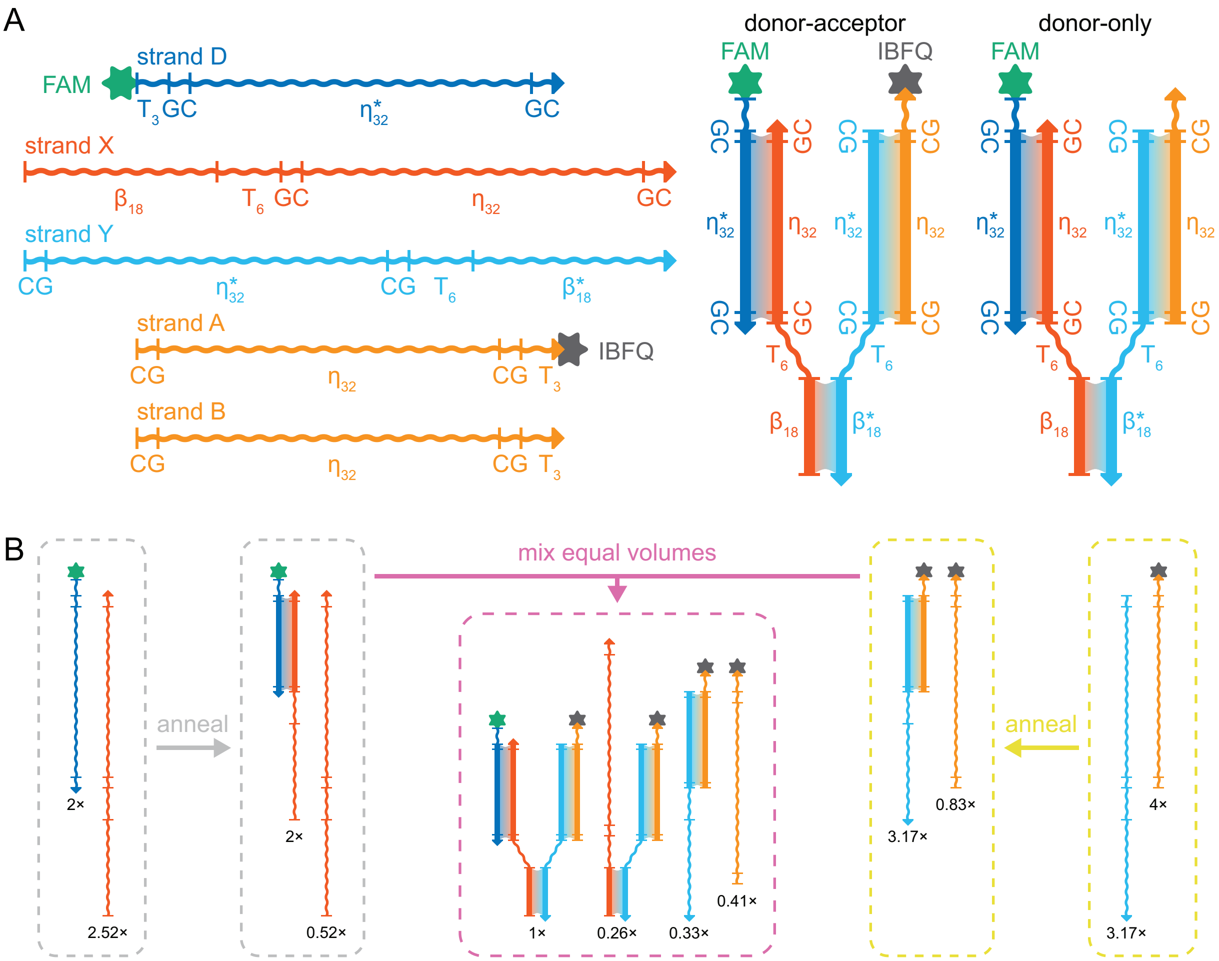

Fig. S2. The mixing/annealing protocol used for preparing DNA constructs, illustrated with respect to homologous tweezers. $\mathbf{(A)}$ Strands D, X, Y, A, and B are required for the donor-acceptor and donor-only variants of homologous tweezers. Nucleotide sequences of domains are given in Table S1, FAM and IBFQ represent fluorescein and Iowa Black FQ, respectively. $\mathbf{(B)}$ Mixing/annealing process of the donor-acceptor variant. Strands comprising the donor- and acceptor-bearing duplexes are annealed separately (where $1\times = 1\ \mu$M). Equal volumes of the annealed products are mixed at room temperature and efficient $\beta : \beta^*$ hybridisation (due to a designed absence of secondary structure in β and β^*) forms tethered duplexes and excess-related complexes that do not affect FRET measurements. Excesses, indicated next to each strand or construct with respect to the target construct, are employed to ensure that all donor-bearing strands are incorporated into complete constructs. The overall ratio of donor- to acceptor-bearing strands is 1:2, and excess acceptor-bearing strands or complexes do not interfere with complete constructs at the measurement concentration (where $1\times = 10$ nM).

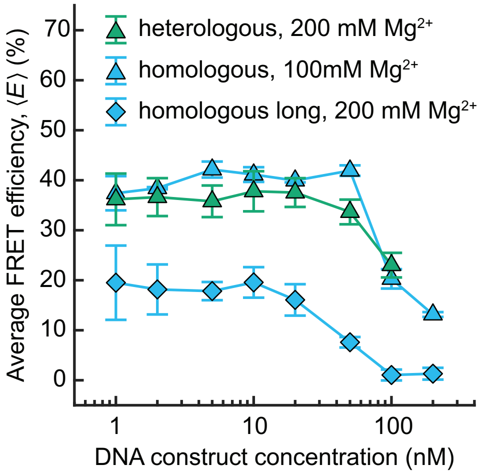

Fig. S3. Concentration-independent FRET confirms the intramolecular nature of FRET from low concentrations of tethered duplexes. Ensemble-averaged FRET efficiency against DNA construct concentration for heterologous (green triangles), homologous (cyan triangles), and homologous, long (cyan diamonds) tweezers. Data shows concentration-independent FRET for $[\mathrm{construct}] \lesssim 50$ nM ($[\mathrm{construct}] \lesssim 20$ nM for long tweezers), confirming the intramolecular nature of FRET for $[\mathrm{construct}] = 10$ nM used throughout this work. For high construct concentrations, FRET is reduced possibly due to intermolecular interactions and/or aggregation. FRET reduction at high concentration may arise from photophysical effects and/or specific structuring (e.g., forcing constructs into elongated, low-FRET configurations) in these potential aggregates. Buffer, 10 mM TRIS with 100 mM NaCl and 100 or 200 mM $MgCl_2$; data points are the mean $\pm$ standard error of 2-3 independent repeats, each independent repeat is the mean of 4 technical repeats.

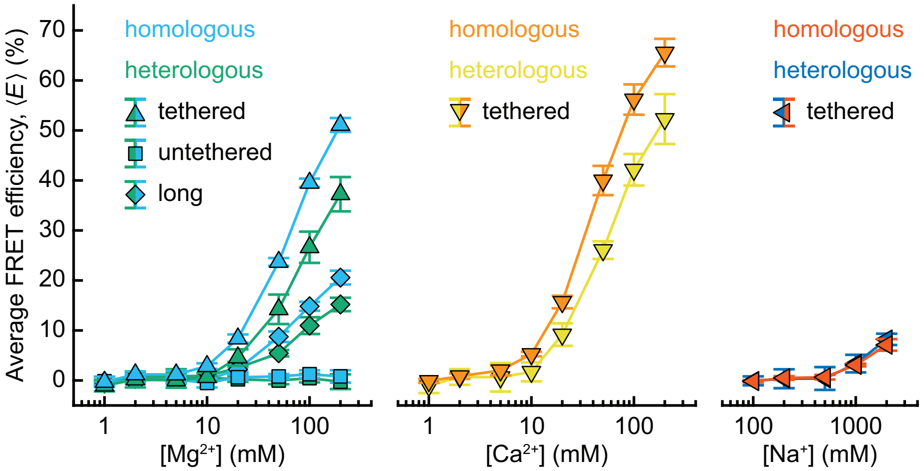

Fig. S4. Emphasising homology-enhanced FRET for tethered constructs in the presence of divalent cations. Figure 1D replot to provide a clearer comparison between the FRET responses of heterologous and homologous constructs in the presence of various concentrations of Mg^{2+}, Ca^{2+}, and Na^{+} cations.

 Andrew Stannard, Ehud Haimov, Jonathan G. Hedley, Yaxuan Xiao, Marco Di Antonio, Gleb Oshanin, Claudia Danilowicz, Mara Prentiss, Lorenzo Di Michele, Alexei A. Kornyshev

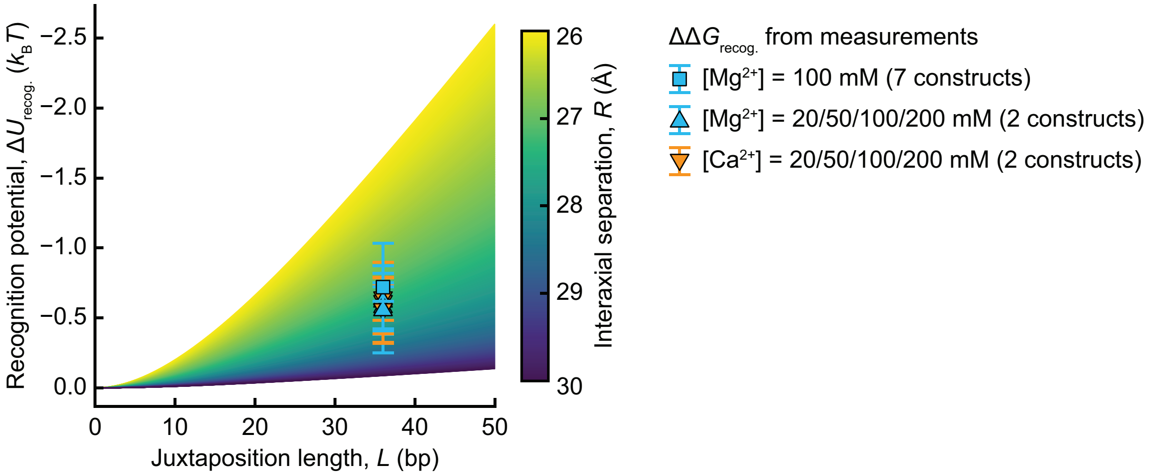

Fig. S5. Comparing a theoretical recognition potential with experimental recognition free energies. Plot of recognition potential, $\Delta U_{\text{recog.}} = U^{\parallel}_{\text{homo.}} - U^{\parallel}_{\text{hetero.}}$, against duplex-duplex juxtaposition length, L, for a range of duplex-duplex interaxial separations, R, with $f_1 = 0.3$, $f_2 = 0.7$, and $\theta = 0.8$, adapted from Ref. (1). Also shown are the experimentally-determined recognition free energies, $\Delta\Delta G_{\text{recog.}}$, from Figures 2B & 3C. The natural agreement is excellent, and possible since $\Delta G = U^{\parallel} + k_{\text{B}}T\ln\left[\Omega\left(U^{\angle}\right)\right]$ where $U^{\angle}$ is independent of homology/heterology (see Section S6), such that $\Delta\Delta G_{\text{recog.}} = \Delta G_{\text{homo.}} - \Delta G_{\text{hetero.}} = \Delta U_{\text{recog.}}$.

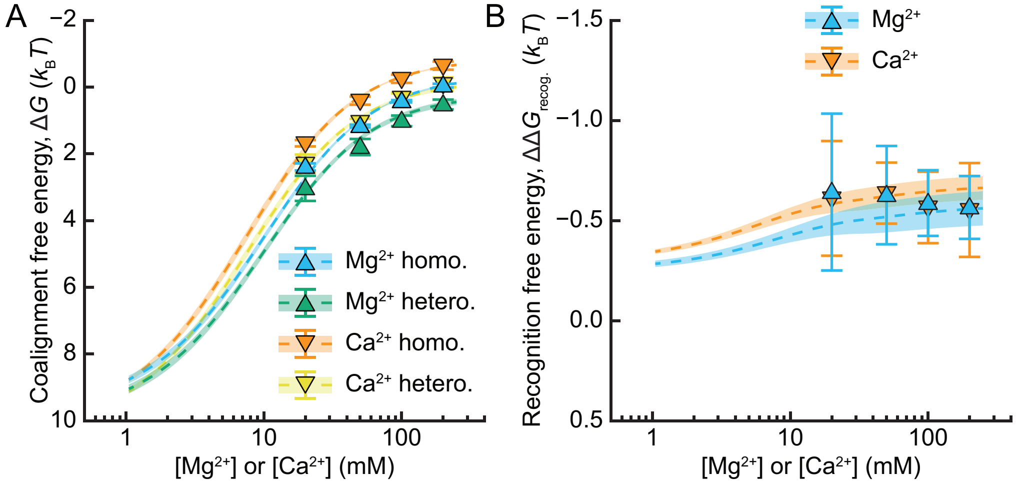

Fig. S6. Extrapolations of model fitting. (**A**) Coalignment and (**B**) recognition free energy against divalent cation concentration, repeated from Figures 3B & 3C, respectively, with model fits extrapolated down to 1 mM. Coalignment free energies drop significantly, as expected, but recognition free energies only lessen slightly.

Supporting Information Text

1. The effect of elastic undulations for long tethered duplexes

Here we describe a theory to approximate the effects of duplex elasticity and rationalise the experimental evidence of lower FRET for longer tethered duplexes, as shown in Figure 1D.

Consider a dsDNA duplex clamped at one end and free to fluctuate at its other end. If the duplex is much longer than a single base-pair, it is well described by the worm-like chain (WLC) model, according to which the average angle θ, between the tangent vectors at the start and end of a duplex of length L is given by (2)

$$\langle \cos\theta \rangle = e^{-L/L_{\mathrm{p}}} \qquad [1]$$

where $L_{\mathrm{p}}(= 50\ \mathrm{nm})$ is the persistence length. This angle defines a thin spherical shell of approximate thickness $L\left[1 - (2/\theta)\sin(\theta/2)\right]$ within which the fluctuating duplex end can be found. The volume of this shell, V, corresponds to the phase space that can be occupied by the fluctuating end. The ratio of such shell volumes between long, 68 bp ($L = 23.1$ nm), and short, 36 bp ($L = 12.2$ nm), duplexes is $V_{68}/V_{36} = 11.7$ (significantly larger than the corresponding surface-area ratio, $A_{68}/A_{36} = 3.57$). For a tethered construct, i.e., two duplexes with fluctuating ends, the extent of the overall available phase space is proportional to V^2.

Now we introduce a set of definitions and assumptions that greatly simplify the calculation of average FRET, $\langle E \rangle$. First, we divide the spherical shell of phase-space points into equal partition volumes, v. The partition volume was chosen as the dome-shaped region enclosed by the solid angle subtended by the FRET markers when their separation is equal to their Förster radius R_{F} and the duplexes are aligned straight. We assume a binary FRET efficiency profile as a function of the FRET marker separation r, given by

$$E = \begin{cases} 1 & r \le R_{\mathrm{F}} \\ 0 & r > R_{\mathrm{F}} \end{cases} \qquad [2]$$

i.e., $E = 1$ within the volume v, and $E = 0$ outside it. We assign a uniform electrostatic energy for all phase space points within v, that of coaligned duplexes. To simplify the analysis further, and since $|U^{\parallel}| \gg |U^{\angle}|$ for all skew angles larger than $\psi^* = 2\arcsin(R_{\mathrm{F}}/2L)$, we assume that at large skew angles $\psi > \psi^*$ the duplexes are non-interacting. Thus, when the phase space is Boltzmann weighted, and assuming $V \gg v$, the average FRET efficiency of a tethered construct is given by

$$\langle E \rangle = \frac{v^2 e^{-U^{\parallel}/k_{\mathrm{B}}T}}{v^2 e^{-U^{\parallel}/k_{\mathrm{B}}T} + V^2}, \qquad [3]$$

where the volumes of the spherical shell and dome-shaped region are given, respectively, by

$$V = 4\pi L^3 \left[1 - \left(\frac{2}{\theta}\right)\sin\left(\frac{\theta}{2}\right)\right] \qquad [4]$$

and

$$v = \frac{\pi L^3}{3}\left[1 - \cos\left(\frac{\psi^*}{2}\right)\right]^2 \left[2 + \cos\left(\frac{\psi^*}{2}\right)\right]. \qquad [5]$$

From Equation 3, the ratio of average FRET efficiencies for 68 bp and 36 bp tethered constructs is given by

$$\frac{\langle E_{68} \rangle}{\langle E_{36} \rangle} = \frac{1 + (V_{36}/v_{36})^2\, e^{U^{\parallel}_{36}/k_{\mathrm{B}}T}}{1 + (V_{68}/v_{68})^2\, e^{U^{\parallel}_{68}/k_{\mathrm{B}}T}}, \qquad [6]$$

where $V_{36}/v_{36} = 93.1$ and $V_{68}/v_{68} = 2100$ can be found using Equations 4 & 5. To explore the behaviour of $\langle E_{68} \rangle / \langle E_{36} \rangle$, for simplicity we consider homologous duplexes, the electrostatic interaction potential of which scales linearly with length, thus $U^{\parallel}_{68} = 1.89 U^{\parallel}_{36}$; Figure S7 shows Equation 6 plot as a function of $U^{\parallel}_{36}$. Since the electrostatic interaction $U^{\parallel}_{36}$ would dramatically change across experiments, depending on the specific ionic conditions, typical values for $U^{\parallel}_{36}$ range from a few positive to a few negative $k_{\mathrm{B}}T$s. In this range, FRET is reduced at least 50% for the 68 bp construct relative to the 36 bp construct, explaining the results reported in Figure 1D. An important characteristic of Equation 6 is that at very small $U^{\parallel}_{36}$, the ratio of FRET efficiencies is proportional to the ratio between the phase-space points of short and long tethered duplexes, which is quite small. However, for very negative $U^{\parallel}_{36}$, tethered duplexes of both lengths would have FRET efficiencies close to 1, which makes the FRET-efficiency ratio converge towards 1, as shown in Figure S7.

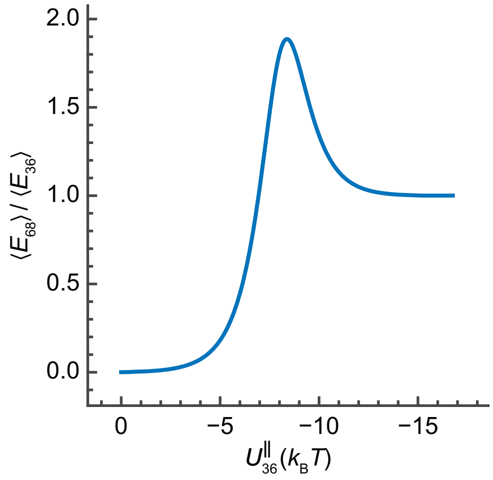

Fig. S7. For weakly-interacting duplexes, the average FRET efficiency, reporting on duplex coalignment, is predicted to decrease with increasing duplex length. Ratio of FRET efficiencies for 68 bp, relative to 36 bp, homologous tethered duplexes, $\langle E_{68}\rangle/\langle E_{36}\rangle$, as a function of coaligned 36 bp duplex-duplex interaction energy, $U_{36}^{\parallel}$.

2. Coalignment free energy from ensemble-averaged FRET efficiency

To derive an expression for the coalignment free energy as a function of ensemble-averaged FRET efficiency, it is assumed that the FRET efficiencies of skewed, low-FRET, states and coaligned, high-FRET, states are $E = 0$ and $E = 1$, respectively. It follows that the ensemble-averaged FRET efficiency, $\langle E \rangle$, gives the fraction of constructs occupying coaligned states, and, thus, is equivalent to the probability of a particular construct occupying a coaligned state, i.e.,

$$p^{\parallel} = \langle E \rangle. \qquad [7]$$

The probability of a particular construct occupying a skewed state is

$$p^{\angle} = 1 - \langle E \rangle \qquad [8]$$

since $p^{\parallel} + p^{\angle} = 1$. The ratio of occupation probabilities is, thus,

$$\frac{p^{\parallel}}{p^{\angle}} = \frac{\langle E \rangle}{1 - \langle E \rangle}. \qquad [9]$$

In addition, these occupancy probabilities are governed by Boltzmann statistics such that

$$p^{\parallel} = \frac{e^{-G^{\parallel}/k_{\mathrm{B}}T}}{Z} \qquad [10]$$

and

$$p^{\angle} = \frac{e^{-G^{\angle}/k_{\mathrm{B}}T}}{Z} \qquad [11]$$

where $G^{\parallel}$ and $G^{\angle}$ are the free energies of coaligned states and skewed states, respectively, and $Z = e^{-G^{\parallel}/k_{\mathrm{B}}T} + e^{-G^{\angle}/k_{\mathrm{B}}T}$ is the partition function. Combining these expressions gives a second expression for the ratio of occupation probabilities,

$$\frac{p^{\parallel}}{p^{\angle}} = e^{-\Delta G/k_{\mathrm{B}}T}, \qquad [12]$$

where $\Delta G = G^{\parallel} - G^{\angle}$ is the coalignment free energy. By equating the two expressions for the ratio of occupation probabilities, and rearranging, an expression for the coalignment free energy in terms of ensemble-averaged FRET efficiency is found,

$$\Delta G = -k_{\mathrm{B}}T \ln\left(\frac{\langle E \rangle}{1 - \langle E \rangle}\right). \qquad [13]$$

3. Cation concentration dependence of the Förster radius

For a donor-acceptor FRET paring, the corresponding Förster radius can be calculated from

$$R_{\mathrm{F}} = \left[\frac{9\ln(10)\,\kappa^2\Phi_{\mathrm{D}}J}{128\pi^5 N_{\mathrm{A}} n^4}\right]^{1/6}, \tag{14}$$

where κ^2 is the orientational factor, Φ_{D} is the donor quantum yield (in the absence of an acceptor), J is the donor-acceptor spectral overlap integral, N_{A} is Avogadro's number, and n is the refractive index of the medium. From the spectral overlapping of fluorescein (FAM) emission and Iowa Black FQ (IBFQ) absorption in pH 8.0 tris-EDTA buffer, IDT determined the FAM-IBFQ Förster radius to be $R_{\mathrm{F}}^0 = 5.79$ nm, the '0' superscript here denotes the absence of metal cations in solution.

At pH 8.0, which our experiments were also performed at, FAM is almost-exclusively present in its dianionic form, which has a near unity quantum yield (0.93 (3)). The introduction of cations to solution from the dissolution of metal salts can, however, result in static (complexation) and/or dynamic (collisional) quenching (4) of FAM to reduce its quantum yield and, thus, its Förster radius with IBFQ. As such, we correct the Förster radius for: (i) the 100 mM monovalent Na^+ cations, present in all solutions for duplex stability; and (ii) the 1-200 mM divalent M^{2+} (Mg^{2+} or Ca^{2+}) cations, which modulate duplex coalignment and homologous recognition. We write the FAM-IBFQ Förster radius as a function of divalent metal cation concentration,

$$R_{\mathrm{F}}(c) = R_{\mathrm{F}}^0 \left[\frac{\Phi_{\mathrm{D}}^{\mathrm{Na}^+}}{\Phi_{\mathrm{D}}^0}\right]^{1/6} \left[\frac{\Phi_{\mathrm{D}}(c)}{\Phi_{\mathrm{D}}^{\mathrm{Na}^+}}\right]^{1/6}, \tag{15}$$

where Φ_{D}^0, $\Phi_{\mathrm{D}}^{\mathrm{Na}^+}$, and $\Phi_{\mathrm{D}}(c)$ are FAM quantum yields in the absence of cations, presence of 100 mM Na^+, and presence of 100 mM Na^+ and c M^{2+}, respectively. Since, in the absence of acceptors, fluorescence is proportional to donor quantum yield, the above ratios can be determined from relative fluorescence intensities of equal FAM concentrations in varying salt conditions, i.e., $\Phi_{\mathrm{D}}^{\mathrm{Na}^+}/\Phi_{\mathrm{D}}^0 = I_{\mathrm{D}}^{\mathrm{Na}^+}/I_{\mathrm{D}}^0$ and $\Phi_{\mathrm{D}}(c)/\Phi_{\mathrm{D}}^{\mathrm{Na}^+} = I_{\mathrm{D}}(c)/I_{\mathrm{D}}^{\mathrm{Na}^+}$ where I_{D}^0, $I_{\mathrm{D}}^{\mathrm{Na}^+}$, and $I_{\mathrm{D}}(c)$ are FAM fluorescence intensities in the absence of cations, presence of 100 mM Na^+, and presence of 100 mM Na^+ and c M^{2+}, respectively.

The first correction accounts for the 100 mM Na^+ cations present in all DNA tweezers solutions. For this, we measured the fluorescence intensity of 10 nM FAM-labelled oligonucleotides in 10 mM tris (pH 8.0) with and without 100 mM NaCl, in addition to buffer solutions without labelled oligonucleotides for background subtraction purposes. FAM-labelled oligonucleotides were used in these measurements as, in the absence of any metal cations in solution, DNA tweezers would be unstable. From 3 technical repeats, we find the relative fluorescence intensities to be $I_{\mathrm{D}}^{\mathrm{Na}^+}/I_{\mathrm{D}}^0 = 0.918\,(\pm 0.015)$ such that $\left[\Phi_{\mathrm{D}}^{\mathrm{Na}^+}/\Phi_{\mathrm{D}}^0\right]^{1/6} = 0.986\,(\pm 0.003)$; the FAM-IBFQ Förster radius in the presence of 100 mM Na^+ is given by

$$R_{\mathrm{F}}^{\mathrm{Na}^+} = R_{\mathrm{F}}^0 \left[\frac{\Phi_{\mathrm{D}}^{\mathrm{Na}^+}}{\Phi_{\mathrm{D}}^0}\right]^{1/6}, \tag{16}$$

such that $R_{\mathrm{F}}^{\mathrm{Na}^+} = 5.71\,(\pm 0.02)$ nm.

For the second, concentration-dependent correction, we re-use the fluorescence measurements of donor-only DNA tweezers which were necessary for determining ensemble-averaged FRET efficiencies. As shown in Figures S8Ai-ii, the fluorescence intensities of donor-only tweezers is homology/heterology independent, as expected, but decreases with increasing divalent cation concentration due to quenching; we pool these data, see Figure S8Aiii. These pooled fluorescence intensities are then normalised by fluorescence intensities in the absence of any divalent M^{2+} cations, but with 100 mM Na^+ cations, to give relative quantum yields, $\Phi_{\mathrm{D}}(c)/\Phi_{\mathrm{D}}^{\mathrm{Na}^+}$, which, raised to the 1/6th power, gives the relative Förster radius, $R_{\mathrm{F}}(c)/R_{\mathrm{F}}^{\mathrm{Na}^+}$, see Figure S8B. Finally, both corrections can be combined to give the FAM-IBFQ Förster radius as a function of divalent metal cation concentration, see Figure S8C.

To capture this trend, we model the concentration-dependent relative Förster radius empirically, by

$$R_{\mathrm{F}}(c)/R_{\mathrm{F}}^{\mathrm{Na}^+} = 1 - \alpha\ln(1+\beta c), \tag{17}$$

and determine α and β from best-fitting to $R_{\mathrm{F}}(c)/R_{\mathrm{F}}^{\mathrm{Na}^+}$ for each divalent ion type, see Figure S8B. We subsequently use these empirical forms of $R_{\mathrm{F}}(c)$ in our thermodynamic expression of coalignment free energy, Equation 24, to fit to experimental data.

Andrew Stannard, Ehud Haimov, Jonathan G. Hedley, Yaxuan Xiao, Marco Di Antonio, Gleb Oshanin, Claudia Danilowicz, Mara Prentiss, Lorenzo Di Michele, Alexei A. Kornyshev

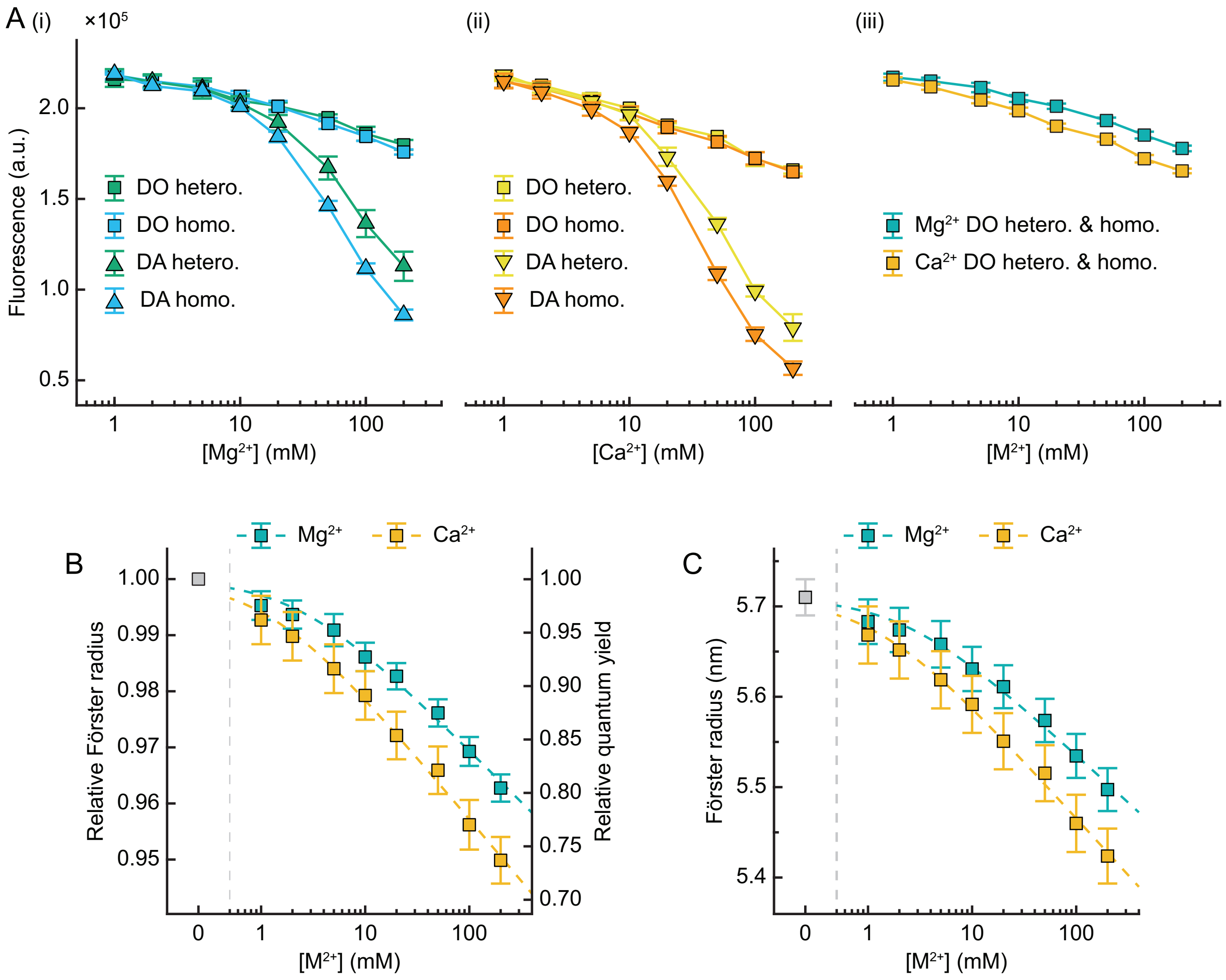

Fig. S8. Cation concentration dependence of the Förster radius. **(A)** Fluorescence intensity against divalent cation concentration for donor-acceptor (DA) and donor-only (DO) heterologous and homologous DNA tweezers for (i) Mg^{2+} and (ii) Ca^{2+} cations; this data is used to determine ensemble-averaged FRET efficiencies in Figure 1D. The fluorescence of donor-only tweezers is clearly homology/heterology independent, as expected, and are (iii) pooled, but decreases with divalent cation concentration due to quenching. The fluorescence intensities of donor-acceptor tweezers decreases more significantly due to coalignment-induced FRET, with enhanced FRET for homologous tweezers due to homologous recognition. **(B)** Relative Förster radius against divalent cation concentration with best fits to the empirical form of Equation 17, where $\alpha = 8.06 \times 10^{-3}$ and $\beta = 0.438\ \mathrm{mM}^{-1}$ for Mg^{2+} and $\alpha = 9.63 \times 10^{-3}$ and $\beta = 0.846\ \mathrm{mM}^{-1}$for Ca^{2+}. The corresponding relative quantum yields are also shown. **(C)** Förster radius against divalent cation concentration, with empirical fitting also shown.

4. Coalignment free energy from coaligned and skewed interaction potentials

To derive an expression of coalignment free energy as a function of coaligned and skewed interaction potentials, consider the duplex-terminating FRET markers to be points on a sphere of radius equal to duplex length, L, with relative angular separation ψ. The FRET efficiency averaged over all duplex-duplex conformations, $\langle E\rangle$, can be obtained by integrating the skew-angle-dependent FRET efficiency, $E(\psi)$, weighted by a Boltzmann factor, over all relative points on a sphere,

$$\langle E\rangle = \frac{\displaystyle\int_0^\pi d\psi\ \sin(\psi)E(\psi)e^{-U(\psi)/k_\mathrm{B}T}}{\displaystyle\int_0^\pi d\psi\ \sin(\psi)e^{-U(\psi)/k_\mathrm{B}T}}, \quad [18]$$

where $U(\psi)$ is skew-angle-dependent interaction potential between duplexes.

Since FRET efficiency decays rapidly (to the sixth power) with marker separation, r, the FRET-efficiency profile can be approximated as a step function, $E(r \le R_\mathrm{F}) = 1$ and $E(r > R_\mathrm{F}) = 0$, where R_F is the Förster radius. In terms of angular separation of points on a sphere of radius L, this approximation yields $E(\psi \le \psi^*) = 1$ and $E(\psi > \psi^*) = 0$, where $\psi^* = 2\arcsin(R_F/2L)$. It follows that the mean FRET efficiency can be approximated as

$$\langle E\rangle \approx \frac{\displaystyle\int_0^{\psi^*} d\psi\ \sin(\psi)e^{-U(\psi)/k_\mathrm{B}T}}{\displaystyle\int_0^{\psi^*} d\psi\ \sin(\psi)e^{-U(\psi)/k_\mathrm{B}T} + \int_{\psi^*}^{\pi} d\psi\ \sin(\psi)e^{-U(\psi)/k_\mathrm{B}T}}. \quad [19]$$

In this work, R_F is the cation-concentration-dependent Förster radius of fluorescein and Iowa Black FQ (see Section S3) and $L = 12.2$ nm is the length of 36 bp duplexes. Thus, ψ^* is reasonably small and, to a first approximation, $U(\psi)$ is effectively constant over the range of relative angles between 0 and ψ^*. Consequently,

$$\int_0^{\psi^*} d\psi\ \sin(\psi)e^{-U(\psi)/k_\mathrm{B}T} \approx \left[1-\cos\left(\psi^*\right)\right] e^{-U^{\parallel}/k_\mathrm{B}T}, \quad [20]$$

where $U^{\parallel}$ is the interaction potential between coaligned duplexes. Applying this approximation gives

$$\langle E\rangle \approx \frac{\left[1-\cos\left(\psi^*\right)\right] e^{-U^{\parallel}/k_\mathrm{B}T}}{\left[1-\cos\left(\psi^*\right)\right] e^{-U^{\parallel}/k_\mathrm{B}T} + \displaystyle\int_{\psi^*}^{\pi} d\psi\ \sin(\psi)e^{-U^{\angle}(\psi)/k_\mathrm{B}T}}, \quad [21]$$

where $U^{\angle}(\psi)$ is the skew-angle-dependent interaction potential between skewed duplexes, which simplifies to

$$\langle E\rangle \approx \frac{e^{-U^{\parallel}/k_\mathrm{B}T}}{e^{-U^{\parallel}/k_\mathrm{B}T} + \Omega\left(U^{\angle}\right)}, \quad [22]$$

where

$$\Omega\left(U^{\angle}\right) = \frac{1}{1-\cos(\psi^*)}\int_{\psi^*}^{\pi} d\psi\ \sin(\psi)e^{-U^{\angle}(\psi)/k_\mathrm{B}T}. \quad [23]$$

Inserting this expression into Equation 13 gives an expression for the coalignment free energy in terms of the coaligned and skewed interactions potentials,

$$\Delta G = U^{\parallel} + k_\mathrm{B}T \ln\left[\Omega\left(U^{\angle}\right)\right]. \quad [24]$$

5. Electrostatic interaction potential of coaligned duplexes

When two dsDNA duplexes are coaligned, their electrostatic interaction potential can be written as

$$U^{\parallel}(R,L;\theta,f_1,f_2) = L\sum_{n=0}^{\infty} a_n(R;\theta,f_1,f_2)\nu_n(R,L), \tag{25}$$

where R is interaxial separation, L is juxtapositional length, θ is overall charge compensation by adsorbed cations, and f_1 and f_2 are fractions of cations adsorbed in the minor and major grooves, respectively. The functions $a_n(R)$ and $\nu_n(L,R)$ are the helical charge harmonics and recognition coefficients, respectively. This summation converges quickly and, for simplicity, is truncated here past the $n=2$ term.

The helical charge harmonics are written as

$$a_0(R;\theta,f_1,f_2) = 2\pi\bar{\sigma}^2\mathcal{W}_{0,0}(0,R,a,b)p_n^2(\theta,f_1,f_2), \tag{26}$$

$$a_{n\geq 1}(R;\theta,f_1,f_2) = 4\pi\bar{\sigma}^2\mathcal{W}_{n,n}(ng,R,a,b)p_n^2(\theta,f_1,f_2), \tag{27}$$

where $\bar{\sigma} = 16.3\ \mu\text{C cm}^{-2}$ is the mean surface charge density of dsDNA, $g = 2\pi/H$ is the reciprocal helical pitch of dsDNA ($H \approx 34$ Å), $a = 10$ Å is the radius of dsDNA, and $b = 6$ Å is the radius of the dielectric core inside dsDNA. $\mathcal{W}_{n,n}$ is a distance-dependent function,

$$\mathcal{W}_{n,m}(q,R,a,b) = w_{nm}(q,R,a,a) + \Xi(m,q)w_{nm}(q,R,a,b)\left[1 + \frac{\Xi(-n,-q)}{\Xi(m,q)} + \Xi(-n,-q)\frac{w_{nm}(q,R,b,b)}{w_{nm}(q,R,a,b)}\right], \tag{28}$$

and p_n is the helical charge pattern,

$$p_n(\theta,f_1,f_2) = \theta[f_1 + f_2(-1)^n] - \cos\left(n\tilde{\phi}_s\right), \tag{29}$$

where $\tilde{\phi}_s \approx 0.4\pi$ is the azimuthal half-width of the minor groove.

For a nonlocal electrolyte, with dielectric function defined as $\varepsilon(k) = \varepsilon_w(k) + \kappa^2/k^2$, where κ^{-1} is the Debye length and $\varepsilon_w(k)$ is the Lorentzian dielectric function of pure water (see Refs. (1, 5)),

$$w_{nm}(q,x,y,z) = \frac{4\pi yz}{\varepsilon}(-1)^m\left[\tilde{g}_1 K_{n-m}(\tilde{Q}_1 x)I_n(\tilde{Q}_1 y)I_m(\tilde{Q}_1 z) + \tilde{g}_2 K_{n-m}(\tilde{Q}_2 x)I_n(\tilde{Q}_2 y)I_m(\tilde{Q}_2 z)\right], \tag{30}$$

where I_n and K_n are the nth order modified Bessel functions of the first and second kind respectively, and

$$\Xi(m,q) = -\frac{a}{b}\,\frac{\mathcal{A}'_m(a,b,q) - \gamma|q|\frac{I'_m(|q|b)}{I_m(|q|b)}\mathcal{A}_m(a,b,q)}{\mathcal{A}'_m(b,b,q) - \gamma|q|\frac{I'_m(|q|b)}{I_m(|q|b)}\mathcal{A}_m(b,b,q)}, \tag{31}$$

where $\gamma = \varepsilon_c/\varepsilon_*$ is the ratio between the dielectric constant in the core, ε_c, and the short-range dielectric constant of water, ε_*, and $I'_n(x) = \partial I_n(x)/\partial x$. The nonlocality of the solvent also appears in $\mathcal{A}_m(x,y,q)$, calculated to be

$$\mathcal{A}_m(x,y,q) = \begin{cases} \frac{4\pi}{\varepsilon}\left[\tilde{g}_1 K_{n-m}(\tilde{Q}_1 x)I_n(\tilde{Q}_1 y) + \tilde{g}_2 K_{n-m}(\tilde{Q}_2 x)I_n(\tilde{Q}_2 y)\right], & x \geq y, \\ \frac{4\pi}{\varepsilon}\left[\tilde{g}_1 K_{n-m}(\tilde{Q}_1 y)I_n(\tilde{Q}_1 x) + \tilde{g}_2 K_{n-m}(\tilde{Q}_2 y)I_n(\tilde{Q}_2 x)\right], & x < y. \end{cases} \tag{32}$$

Note that when computing $\mathcal{A}'_m = \partial\mathcal{A}_m/\partial y$, the correct case above must be chosen carefully. The characteristic lengths in these functions are $\tilde{Q}_i = \sqrt{Q_i^2 + q^2}$, where

$$Q_1 = \frac{1}{\sqrt{2}}\sqrt{\xi\kappa^2 + \frac{1}{\Lambda^2}\left[1 + \sqrt{(1-2\kappa\Lambda+\xi\kappa^2\Lambda^2)(1+2\kappa\Lambda+\xi\kappa^2\Lambda^2)}\right]}, \tag{33}$$

$$Q_2 = \frac{1}{\sqrt{2}}\sqrt{\xi\kappa^2 + \frac{1}{\Lambda^2}\left[1 - \sqrt{(1-2\kappa\Lambda+\xi\kappa^2\Lambda^2)(1+2\kappa\Lambda+\xi\kappa^2\Lambda^2)}\right]}, \tag{34}$$

where $\xi = \varepsilon/\varepsilon_*$ is the ratio between bulk, ε, and short-range, ε_*, dielectric constants, and Λ is the characteristic length of nonlocal water polarisation correlations. The coefficients $\tilde{g}_i$ are then given by

$$\tilde{g}_1 = \frac{\xi\Lambda^2\tilde{Q}_{1,q=0}^2 - 1}{\sqrt{(1-2\kappa\Lambda+\xi\kappa^2\Lambda^2)(1+2\kappa\Lambda+\xi\kappa^2\Lambda^2)}}, \tag{35}$$

$$\tilde{g}_2 = \frac{1 - \xi\Lambda^2\tilde{Q}_{2,q=0}^2}{\sqrt{(1-2\kappa\Lambda+\xi\kappa^2\Lambda^2)(1+2\kappa\Lambda+\xi\kappa^2\Lambda^2)}}. \tag{36}$$

The recognition coefficients contain information about the sequences of the interacting dsDNA duplexes. For any two duplexes with random sequences (heterologous), distortions in the helical pitch lead to a dephasing of their helical structure over the coherence length $\lambda_c \approx 100$ Å (6). Accounting for this, the recognition coefficients are defined as

$$\nu_n(R, L) = \frac{2\lambda_\mathrm{c}}{n^2 L}\left(1 - e^{-n^2 L/2\lambda_\mathrm{c}}\right)\cos[n\delta\phi^*(R, L)], \qquad [37]$$

where $\delta\phi^*(R)$ is the optimum relative azimuthal orientation of the two duplexes (i.e. the $\delta\phi$ that minimises the electrostatic energy) (1). When truncating the electrostatic potential after the $n = 2$ term, this angle can be approximated as

$$\delta\phi^*(R, L) \approx \begin{cases} \pm\arccos\left[\dfrac{|a_1(R)|}{a_2(R)}\dfrac{1 - e^{-L/2\lambda_\mathrm{c}}}{1 - e^{-2L/\lambda_\mathrm{c}}}\right], & \dfrac{|a_1(R)|}{a_2(R)}\dfrac{1 - e^{-L/2\lambda_\mathrm{c}}}{1 - e^{-2L/\lambda_\mathrm{c}}} \leq 1, \\ 0, & \dfrac{|a_1(R)|}{a_2(R)}\dfrac{1 - e^{-L/2\lambda_\mathrm{c}}}{1 - e^{-2L/\lambda_\mathrm{c}}} > 1. \end{cases} \qquad [38]$$

For homologous duplexes, $\lambda_\mathrm{c} \to \infty$, as their sequences are correlated over their entire juxtapositional length; in this case, the recognition coefficients simply become

$$\nu_{n,\mathrm{homo.}}(R) = \lim_{\lambda_\mathrm{c}\to\infty} \nu_n(R, L) = \cos[n\delta\phi^*_{\mathrm{homo.}}(R)] \qquad [39]$$

where

$$\delta\phi^*_{\mathrm{homo.}}(R) = \begin{cases} \pm\arccos\left(\dfrac{|a_1(R)|}{4a_2(R)}\right), & |a_1(R)| \leq 4a_2(R), \\ 0, & |a_1(R)| > 4a_2(R). \end{cases} \qquad [40]$$

Note that the use of the Debye–Hückel approximation in this context is justified in what is effectively a Debye–Bjerrum sense. Strong adsorption of multivalent cations onto dsDNA substantially reduces the net charge of the duplex. Under such conditions, the residual electrostatic field is sufficiently weak that a linearized Poisson–Boltzmann (Debye–Hückel) description of the surrounding ionic atmosphere becomes a reasonable approximation (7).

It is non-trivial, however, to assume that the Debye–Hückel approximation remains valid for the inherently nonlocal electrostatics around dsDNA. A recent study combining a field-theoretical framework with all-atom molecular dynamics simulations, that explicitly included water molecules, computed the electric field surrounding dsDNA in solutions of mono- and di-valent salts (8). That work directly compared the computed electric field with predictions of a nonlocal Debye–Hückel description and found no significant differences at distances greater than ∼5-6 Å from the dsDNA surface. Since the interactions considered in our study occur at separations beyond this range, these results support the applicability of the Debye–Hückel approximation in the present case.

6. Electrostatic interaction potential of skewed duplexes

Compared to coaligned duplexes, the nonlocal electrostatic interaction potential for skewed duplexes, $U^{\angle}$ is substantially more complicated. Fortunately, here, the theoretical framework only requires consideration of skew angles $\psi > \psi^*$, when only an effective duplex length, $\sim\kappa^{-1}/|\sin\psi|$, which is much smaller than the helical coherence length, λ_{c}, needs consideration. This allows two major simplifications:

(i) duplexes can be considered as ideal double helices as there is no significant difference between homologous and heterologous duplexes for $\psi > \psi^*$; and

(ii) duplexes can be extended to infinite length without changing the expression for interaction potential.

To further simplify calculations, fully-symmetric skewed configurations are considered. Thus, expanding on (ii), both duplexes can be analytically continued beyond their connection point, ultimately to infinite length in both directions. This doubles the interaction potential, which can simply be corrected by halving the final analytical expression.

Following these considerations, the calculation is reduced to finding the interaction potential between two infinitely-long, ideal double helices at skew angle ψ. This has previously been calculated for a local response model (9), assuming the dielectric constant to be $\varepsilon \approx 80$ everywhere. Using concepts presented in Ref. (1) for nonlocal solvent electrostatics of double helices in coaligned juxtapositions, the interaction potential derived in Ref. (9) is directly extended to accommodate the same nonlocal dielectric response as Ref. (1). Thus, half the electrostatic interaction potential between of two infinitely-long, ideal double helices at a skew angle ψ, embedded within a nonlocal electrolyte can be written as

$$U^{\angle}(R,\psi;\theta,f_1,f_2) = \frac{1}{|\sin\psi|}\sum_{n,m=-\infty}^{\infty} a^{\angle}_{nm}(R,\psi,\theta,f_1,f_2). \qquad [41]$$

Here, the skewed helical harmonics are given by

$$\begin{aligned} a^{\angle}_{nm}(R,\psi;\theta,f_1,f_2) = \frac{\ell_{\mathrm{B}}^{(0)} k_{\mathrm{B}}T}{\pi a^2 h_{\mathrm{r}}^2}\cos(n\phi_1 - m\phi_2)p_n(\theta,f_1,f_2)p_{-m}(\theta,f_1,f_2) \\ \times\Big\{a^2\mathcal{S}_{nm}(R,\psi,a,a) \\ +ab[\Xi(m)\mathcal{S}_{nm}(R,\psi,a,b)+\Xi(-n)\mathcal{S}_{nm}(R,\psi,b,a)] \\ +b^2\Xi(-n)\Xi(m)\mathcal{S}_{nm}(R,\psi,b,b)\Big\} \end{aligned} \qquad [42]$$

where, for brevity, $\Xi(n) \equiv \Xi(n, ng)$, and

$$\mathcal{S}_{nm}(R,\psi,x,y) = \frac{4\pi}{\varepsilon\Lambda^2}\left(\frac{1}{\tilde{Q}^2_{2,m}-\tilde{Q}^2_{1,m}}\right)\left[s^{(1)}_{nm}(R,\psi,x,y) - s^{(2)}_{nm}(R,\psi,x,y)\right], \qquad [43]$$

where

$$\begin{aligned} s^{(i)}_{nm}(R,\psi,x,y) = \pi(-1)^{n+m}(1-\xi\Lambda^2 Q_i^2)e^{-R\sqrt{u^2_{nm}(\psi)+\tilde{Q}^2_{i,m}}} \\ \times\frac{I_n(\tilde{Q}_{i,n}x)I_m(\tilde{Q}_{i,m}y)}{\sqrt{u^2_{nm}(\psi)+\tilde{Q}^2_{i,m}}}\left[\frac{\sqrt{u^2_{mn}(\psi)+\tilde{Q}^2_{i,n}}+u_{mn}(\psi)}{\sqrt{u^2_{mn}(\psi)+\tilde{Q}^2_{i,n}}-u_{mn}(\psi)}\right]^{n/2}\left[\frac{\sqrt{u^2_{nm}(\psi)+\tilde{Q}^2_{i,m}}+u_{nm}(\psi)}{\sqrt{u^2_{nm}(\psi)+\tilde{Q}^2_{i,m}}-u_{nm}(\psi)}\right]^{m/2}, \end{aligned} \qquad [44]$$

and $\tilde{Q}^2_{i,n} = Q_i^2 + n^2g^2$, and $u_{nm}(\psi) = (ng - mg\cos\psi)/\sin\psi$. In Equation 42, $h_{\mathrm{r}} = 3.4$ Å is the helical rise of a base pair, and $\ell_{\mathrm{B}}^{(0)} \approx 560$ Å is the vacuum Bjerrum length. When considering skewed duplexes, in contrast to the coaligned case, we can no longer define the relative azimuthal orientation, $\delta\phi$, and instead must consider the rotation of each duplex independently, as ϕ_1 and ϕ_2. For all calculations, we assume that at any angle ψ, the duplexes rotate instantaneously about their individual axes to minimise their energy. Thus, when the integral in Equation 23 is computed numerically, $U^{\angle}$ is first minimised with respect to both ϕ_1 and ϕ_2, for every value of $\psi \in [\psi^*, \pi]$.

7. Fitting procedure

Our experimental observable is the coalignment free energy, ΔG, obtained from the ensemble-averaged FRET efficiency via Equation 13. The model prediction is given by

$$\Delta G_{\mathrm{model}}(\vartheta; c) = U^{\parallel}(\vartheta; c) + k_{\mathrm{B}}T \ln\left[U^{\angle}(\vartheta; c)\right], \tag{45}$$

with component terms defined in Sections S5 & S6 and $\vartheta = (R, f_2, 1/K_{\mathrm{d}})$ denoting the vector of shared fitting parameters. We perform a single joint fit to the two, homologous (h) and heterologous (nh), experimental datasets. For experimental concentrations c_i $(i = 1, ...N)$, let the experimentally-inferred values be $\Delta G_i^{(\mathrm{h})}$ and $\Delta G_i^{(\mathrm{nh})}$ with reported standard errors $\sigma_i^{(\mathrm{h})}$ and $\sigma_i^{(\mathrm{nh})}$. The best-fit parameters are obtained by minimising a joint weighted sum of squared, error-normalised residuals,

$$\mathcal{L}(\vartheta) = \sum_{i=1}^{N} w_i^{(\mathrm{h})} \left(\frac{\Delta G_{\mathrm{model}}^{(\mathrm{h})}(\vartheta; c_i) - \Delta G_i^{(\mathrm{h})}}{\sigma_i^{(\mathrm{h})}} \right)^2 + \sum_{i=1}^{N} w_i^{(\mathrm{nh})} \left(\frac{\Delta G_{\mathrm{model}}^{(\mathrm{nh})}(\vartheta; c_i) - \Delta G_i^{(\mathrm{nh})}}{\sigma_i^{(\mathrm{nh})}} \right)^2. \tag{46}$$

The weights w_i are fixed emphasis factors, used only to slightly down-/up-weight specific points, and do not affect the qualitative conclusions. Denoting the nominal fit by ϑ_0, we find

$$\vartheta_0^{\mathrm{Mg}^{2+}} = (27.913\ \text{Å}, 0.991, 0.0616\ \mathrm{mM}^{-1}), \quad \vartheta_0^{\mathrm{Ca}^{2+}} = (27.654\ \text{Å}, 0.999, 0.0834\ \mathrm{mM}^{-1}). \tag{47}$$

To quantify local statistical uncertainty of the fitted parameters from the joint fit, we write $\mathcal{L}(\vartheta) = \|r(\vartheta)\|^2$ in terms of the whitened residual vector, $r(\vartheta)$, whose components are

$$r_i^{(\mathrm{h})}(\vartheta) = \sqrt{w_i^{(\mathrm{h})}} \frac{\Delta G_{\mathrm{model}}^{(\mathrm{h})}(\vartheta; c_i) - \Delta G_i^{(\mathrm{h})}}{\sigma_i^{(\mathrm{h})}}, \quad r_i^{(\mathrm{nh})}(\vartheta) = \sqrt{w_i^{(\mathrm{nh})}} \frac{\Delta G_{\mathrm{model}}^{(\mathrm{nh})}(\vartheta; c_i) - \Delta G_i^{(\mathrm{nh})}}{\sigma_i^{(\mathrm{nh})}}. \tag{48}$$

We then compute the Jacobian, $J = \partial r / \partial \vartheta^{\mathrm{T}}$, at the optimum $\hat{\vartheta} = \vartheta_0$ and use the Gauss-Newton approximation for nonlinear least squares,

$$\Sigma_\vartheta \approx \left(J^{\mathrm{T}} J\right)^{-1}, \quad \sigma_{\mathrm{fit}}(\vartheta_j) = \sqrt{[\Sigma_\vartheta]_{jj}}. \tag{49}$$

For the nominal fit, this yields 1σ fitting uncertainties of

$$\sigma_{\mathrm{fit}}^{\mathrm{Mg}^{2+}}(\vartheta) \approx (0.108\ \text{Å}, 0.021, 0.0021\ \mathrm{mM}^{-1}), \quad \sigma_{\mathrm{fit}}^{\mathrm{Ca}^{2+}}(\vartheta) \approx (0.066\ \text{Å}, 0.015, 0.0025\ \mathrm{mM}^{-1}). \tag{50}$$

For uncertainty bands in ΔG_{h}, ΔG_{nh}, and $\Delta\Delta G_{\mathrm{recog.}}$, we use $\hat{\vartheta}$ and Σ_ϑ to approximate the local sampling distribution as

$$\vartheta \sim \mathcal{N}\left(\hat{\vartheta}, \Sigma_\vartheta\right), \tag{51}$$

and enforce the following constraints on the parameters via rejection sampling:

$$R > 0, \quad 0 < f_2 < 1, \quad 1/K_{\mathrm{d}} > 0. \tag{52}$$

In practice, this effectively means sampling from a truncated multivariate normal distribution. For each accepted draw from the distribution, $\vartheta^{(s)}$, we calculate

$$\Delta G_{\mathrm{h}}^{(s)}(c), \quad \Delta G_{\mathrm{nh}}^{(s)}(c), \quad \Delta\Delta G_{\mathrm{recog.}}^{(s)}(c) = \Delta G_{\mathrm{h}}^{(s)}(c) - \Delta G_{\mathrm{nh}}^{(s)}(c). \tag{53}$$

This yields an ensemble of curves $\{\Delta G_{\mathrm{h}}^{(s)}(c)\}_{s=1}^{S}$, $\{\Delta G_{\mathrm{nh}}^{(s)}(c)\}_{s=1}^{S}$, and $\{\Delta\Delta G_{\mathrm{recog.}}^{(s)}(c)\}_{s=1}^{S}$ that represent how predictions change under statistically plausible perturbations of the fitted parameters. For each concentration in our predicted curve, c_j, we calculate ΔG_{h}, ΔG_{nh} and $\Delta\Delta G_{\mathrm{recog.}}$, and compute the empirical 16th and 84th percentiles across the ensemble:

$$\Delta G_{\mathrm{h,low}}(c_j) = Q_{0.16}\left(\{\Delta G_{\mathrm{h}}^{(s)}(c)\}_s\right), \quad \Delta G_{\mathrm{h,high}}(c_j) = Q_{0.84}\left(\{\Delta G_{\mathrm{h}}^{(s)}(c)\}_s\right), \tag{54}$$

then plot the best-fit curve $\Delta G_{\mathrm{h}}\left(\hat{\vartheta}; c\right)$ as the dashed line, and shade the region $[\Delta G_{\mathrm{h,low}}, \Delta G_{\mathrm{h,high}}]$ (and analogously for ΔG_{nh} and $\Delta\Delta G_{\mathrm{recog.}}$). This yields a 68% ($\sim 1\sigma$) pointwise confidence band around the best-fit prediction, reflecting statistical uncertainty in the fitted parameters propagated through our model.

8. Relaxing the step-function FRET approximation

In sections S2 and S4, the coaligned and skewed sectors are distinguished using a step-function approximation to the FRET efficiency, such that $E = 1$ for $\psi \leq \psi^*$ and $E = 0$ for $\psi > \psi^*$. This approximation underlies the extraction of coalignment free energies from the experimentally measured ensemble-averaged FRET efficiencies. Since the true FRET efficiency is continuous, skewed configurations can in principle make a small nonzero contribution to the measured FRET. Here we estimate the size of that contribution and assess its effect on the inferred free energies.

We replace the step-function approximation by the continuous FRET efficiency

$$E(r;c) = \frac{1}{1 + \left(\dfrac{r}{R_F(c)}\right)^6}, \quad [55]$$

with $r(\psi) = 2L\sin(\psi/2)$ and with the concentration dependent Förster radius $R_F(c)$ estimated as described in Section S3. The full predicted mean FRET is then

$$\langle E\rangle(c) = \frac{\displaystyle\int_0^\pi d\psi\ \sin(\psi)E(\psi;c)e^{-U(\psi;c)/k_BT}}{\displaystyle\int_0^\pi d\psi\ \sin(\psi)e^{-U(\psi;c)/k_BT}} \quad [56]$$

where

$$U(\psi;c) = \begin{cases} U_\parallel(c), & \psi \leq \psi^*(c), \\ U_\angle(\psi;c), & \psi > \psi^*(c). \end{cases} \quad [57]$$

Here, $U_\parallel$ and $U_\angle$ are the interaction potentials defined in Sections S5 and S6. As in the main thermodynamic analysis, these quantities are evaluated using the fitted parameters R, f_2 and K_d (Section S7), together with the concentration-dependent Förster radius $R_F(c)$, which relates directly to the threshold angle $\psi^*(c)$ as described in Section S3.

The contribution of skewed configurations to the total predicted FRET can then be written as

$$\langle E\rangle_\angle(c) = \frac{\displaystyle\int_{\psi^*(c)}^\pi d\psi\ \sin(\psi)E(\psi;c)e^{-U_\angle(\psi;c)/k_BT}}{\displaystyle\int_0^\pi d\psi\ \sin(\psi)e^{-U(\psi;c)/k_BT}}. \quad [58]$$

This quantity measures the part of the overall ensemble-averaged FRET signal arising from the skewed sector. However, for re-extracting free energies from the experimental FRET efficiencies, the more relevant quantity is the mean FRET within the skewed sector itself,

$$\bar{E}_\angle(c) = \frac{\displaystyle\int_{\psi^*(c)}^\pi d\psi\ \sin(\psi)E(\psi;c)e^{-U_\angle(\psi;c)/k_BT}}{\displaystyle\int_{\psi^*(c)}^\pi d\psi\ \sin(\psi)e^{-U_\angle(\psi;c)/k_BT}}. \quad [59]$$

The distinction is that $\langle E\rangle_\angle(c)$ is the skewed-sector contribution to the total signal, whereas $\bar{E}_\angle(c)$ is the conditional mean FRET of skewed configurations. The two are related by

$$\langle E\rangle_\angle(c) = p_\angle(c)\bar{E}_\angle(c), \quad [60]$$

where

$$p_\angle(c) = \frac{\displaystyle\int_{\psi^*(c)}^\pi d\psi\ \sin(\psi)e^{-U_\angle(\psi;c)/k_BT}}{\displaystyle\int_0^\pi d\psi\ \sin(\psi)e^{-U(\psi;c)/k_BT}}. \quad [61]$$

To determine how a finite skewed-state FRET contribution affects the experimentally inferred free energies, we write the total FRET efficiency as

$$\langle E\rangle = p_\parallel\bar{E}_\parallel + (1-p_\parallel)\bar{E}_\angle, \quad [62]$$

where $p_\parallel$ is the fraction of the population in coaligned states, and $\bar{E}_\parallel$ and $\bar{E}_\angle$ are the mean FRET efficiences of the coaligned and skewed sectors, respectively. Taking $\bar{E}_\parallel = 1$, as in the analysis in the main text, gives

$$p_\parallel = \frac{\langle E\rangle - \bar{E}_\angle}{1-\bar{E}_\angle}, \quad p_\angle = 1 - p_\parallel = \frac{1-\langle E\rangle}{1-\bar{E}_\angle}. \quad [63]$$

The ratio of occupation probabilities is therefore

$$\frac{p_\parallel}{p_\angle} = \frac{\langle E\rangle - \bar{E}_\angle}{1-\langle E\rangle}. \quad [64]$$

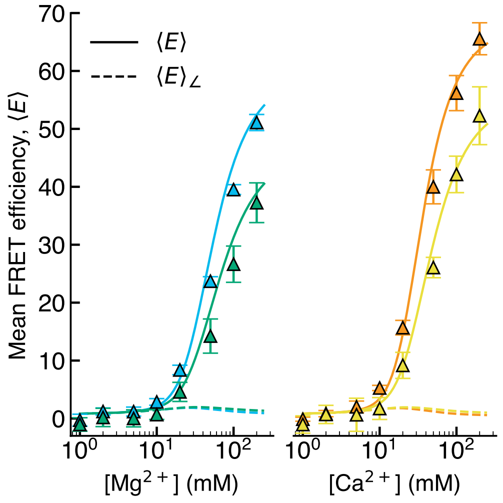

Fig. S9. FRET contribution of the skewed state for a continuous FRET efficiency. Solid curves show the predicted mean FRET, $\langle E \rangle = \langle E \rangle_{\parallel} + \langle E \rangle_{\angle}$, and dashed curves show the contribution from skewed states, $\langle E \rangle_{\angle}$, computed using the continuous FRET efficiency together with the analytical electrostatic interaction energies $U_{\parallel}(c)$ and $U_{\angle}(\psi; c)$, evaluated at the parameter values R, f_2 and K_d obtained from fitting our step approximation. Left: Mg^{2+}, Right: Ca^{2+}. Blue/orange curves correspond to homologous constructs; green/yellow curves correspond to heterologous constructs.

Assuming, ss in Section S1, the occupation probabilities are governed by Boltzmann statistics,

$$\frac{p_{\parallel}}{p_{\angle}} = \exp\left(-\frac{\Delta G}{k_B T}\right), \qquad [65]$$

where $\Delta G = G_{\parallel} - G_{\angle}$ is the coalignment free energy, we obtain

$$\Delta G = -k_B T \ln\left(\frac{\langle E \rangle - \bar{E}_{\angle}}{1 - \langle E \rangle}\right) \qquad [66]$$

In the limit $\bar{E}_{\angle} = 0$, this reduces to Eq. (7) of Section S1.

The resulting curves for $\langle E \rangle_{\angle}(c)$, shown in Figure S9, demonstrate that the continuous Förster efficiency produces a small but nonzero contribution from skewed states for both Mg^{2+} and Ca^{2+}. This contribution remains weak across the concentration range considered; the dominant signal at higher divalent-cation concentrations therefore still arises from the coaligned state, consistent with the measured trends in Fig. 1D and the fitted free-energy curves in Fig. 3B of the main text.

The corresponding values of $\bar{E}_{\angle}(c)$ can then be used to re-extract the free energies from the measured FRET efficiencies using Eq. (66), rather than the step-function expression in Section S1. The resulting curves, shown in Fig S10, show that relaxing the step-function approximation produces only modest changes in the inferred free energies overall. The largest visible deviation appears at the low-concentration end of the heterologous Mg^{2+} curve, where the experimental uncertainties are also greatest. Importantly, the inferred recognition free energy remains robust: allowing for a finite skewed-state contribution does not reduce the recognition effect and, if anything, slightly increases the magnitude of $\Delta\Delta G$. Thus, the simpler step-function treatment used in the main text already captures the effect at the correct scale.

Overall, while the full continuous-FRET treatment is more complete, it substantially complicates the analysis while producing only a limited quantitative correction. We therefore conclude that the simpler step-function approximation used in the main text is justified for the purpose of extracting the recognition free energy.

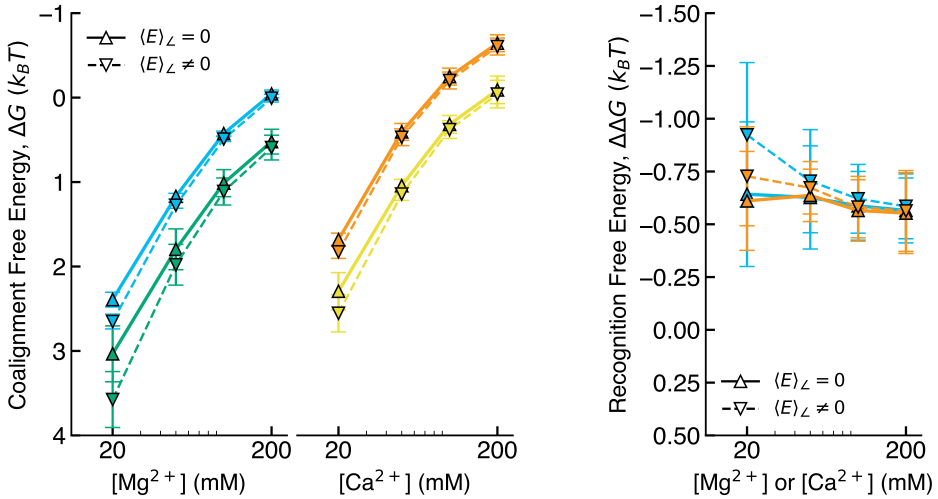

Fig. S10. Effect of relaxing the step-function approximation for the FRET efficiency on the extracted free energies. Left and middle panels show the inferred coalignment free energies, ΔG, for Mg^{2+} and Ca^{2+}, respectively; in these panels, blue and orange denote homologous sequences, while green and yellow denote heterologous sequences. The right panel shows the corresponding recognition free energies, $\Delta\Delta G$, with blue for Mg^{2+} and orange for Ca^{2+}. Solid curves denote the values obtained under the approximation $\langle E \rangle_\angle = 0$ used in the main text, while dashed curves show the results when this approximation is relaxed to allow a finite FRET contribution from skewed configurations.

References

1. E Haimov, JG Hedley, AA Kornyshev, Nonlocal structural effects of water on DNA homology recognition. *J. Physics: Condens. Matter* **36**, 40LT01 (2024).
2. M Rubinstein, RH Colby, *Polymer Physics.* (Oxford University Press), (2003).
3. R Sjöback, J Nygren, M Kubista, Absorption and fluorescence properties of fluorescein. *Spectrochimica Acta Part A: Mol. Biomol. Spectrosc.* **51**, L7–L21 (1995).
4. JR Lakowicz, *Principles of Fluorescence Spectroscopy.* (Springer, New York), 3rd edition, (2006).
5. AA Kornyshev, Non-local dielectric response of a polar solvent and Debye screening in ionic solution. *J. Chem. Soc., Faraday Trans. 2* **79**, 651–661 (1983).
6. A Wynveen, DJ Lee, AA Kornyshev, S Leikin, Helical coherence of DNA in crystals and solution. *Nucleic Acids Res.* **36**, 5540–5551 (2008).
7. AA Kornyshev, DJ Lee, S Leikin, A Wynveen, Structure and interactions of biological helices. *Rev. Mod. Phys.* **79**, 943–996 (2007).
8. JG Hedley, K Coshic, A Aksimentiev, AA Kornyshev, Electric Field of DNA in Solution: Who Is in Charge? *Phys. Rev. X* **14**, 031042 (2024).
9. AA Kornyshev, S Leikin, Electrostatic interaction between long, rigid helical macromolecules at all interaxial angles. *Phys. Rev. E* **62**, 2576–2596 (2000).
